# A synopsis of *Salvatoria* McIntosh, 1885 (Annelida: Syllidae: Exogoninae) from Brazilian coastal and oceanic waters

**DOI:** 10.1371/journal.pone.0250472

**Published:** 2021-05-05

**Authors:** Rodolfo Leandro Nascimento, Marcelo Veronesi Fukuda, Karla Paresque, João Miguel de Matos Nogueira, Paulo Cesar de Paiva

**Affiliations:** 1 Laboratório de Polychaeta, Departamento de Zoologia, Instituto de Biologia, Universidade Federal do Rio de Janeiro, Rio de Janeiro, Rio de Janeiro, Brazil; 2 Programa de Pós–graduação em Biodiversidade e Biologia Evolutiva, Instituto de Biologia, Universidade Federal do Rio de Janeiro, Rio de Janeiro, Rio de Janeiro, Brazil; 3 Museu de Zoologia, Universidade de São Paulo, São Paulo, São Paulo, Brazil; 4 Laboratório de Bentologia, Instituto de Ciências Biológicas e da Saúde, Universidade Federal de Alagoas, Maceió, Alagoas, Brazil; 5 Laboratório de Poliquetologia (LaPol), Departamento de Zoologia, Instituto de Biociências, Universidade de São Paulo, São Paulo, São Paulo, Brazil; CIIMAR Interdisciplinary Centre of Marine and Environmental Research of the University of Porto, PORTUGAL

## Abstract

We compiled the records for the genus *Salvatoria* from Brazilian coastal and oceanic habitats, collected by several projects along the years. Here we present 12 species, eight of which already reported–*S*. *breviarticulata* **comb. nov.**, *S*. *clavata*, *S*. *euritmica*, *S*. *heterocirra*, *S*. *limbata*, *S*. *longiarticulata* **comb. nov.**, *S*. *neapolitana* and *S*. cf. *nitidula*–with comments regarding the confidence of some of these records. We also describe three new species, *S*. *marielleae* **n. sp.** and *Salvatoria nitiduloides* **n. sp.**, based on material from Fernando de Noronha and Trindade islands, off the Northeastern Brazilian coast, and *S*. *ypsiloides* **n. sp.**, from Fernando de Noronha and also, Campos Basin, off Southeastern Brazilian coast, in depths down to 970 m. Finally, we report a probably undescribed species, *Salvatoria* sp., represented by only one specimen lacking median antenna, preventing us to proceed with further identification properly. A dichotomous identification key and a comparative table with morphological data of specimens belonging to these species are also provided.

## Introduction

The family Syllidae Grube, 1850 [1] is one of the most diverse of Annelida, with 1100 valid species, distributed in 79 genera [2] and five subfamilies [3]: Anoplosyllinae Aguado & San Martín, 2009 [4], Autolytinae Langerhans, 1879 [5], Eusyllinae Malaquin, 1893 [6], Exogoninae Langerhans, 1879 [5], and Syllinae Grube, 1850 [1], in addition to some *incertae sedis* genera. Complete introductions on the family are provided in [3, 7, 8]. Currently, approximately 153 species of syllids are reported from Brazilian waters, most of them from coastal habitats, especially off the Southeastern coast [9–12].

The subfamily Exogoninae is a monophyletic group, however, some of its genera, such as *Salvatoria* McIntosh, 1885 [13], are not [14]. The most recent phylogenetic hypothesis recovered *Salvatoria* as a paraphyletic group, based on molecular and morphological data [8]. The presence of 2 pairs of peristomial cirri led these animals to be initially described in the genus *Brania* Quatrefages, 1866 [15]. However, San Martín [16] recognized consistent morphological differences between two groups of species within *Brania*, corresponding to different brooding modes–ventral brooding of eggs and embryos, and dorsal brooding of eggs–, processes that apparently have strong phylogenetic signal [8, 13, 17]; the author, therefore, described the genus *Pseudobrania* San Martin, 1984 [16] to accommodate the species of the second group. It was later recognized that the name *Grubeosyllis* Verrill, 1900 [18] had priority over *Pseudobrania* [19] and, further, that *Salvatoria* McIntosh, 1885 has priority over *Grubeosyllis* Verrill, 1900 [3, 7]. Currently, the genus comprises 31 valid species distributed worldwide, including the three new ones described herein [20–22].

Up to this paper, eight species of the genus had been reported in Brazilian waters. Among these species, only *Salvatoria breviarticulata* (Nogueira, San Martín & Amaral, 2001) [21] **comb. nov.** (as *Grubeosyllis breviarticulata*) and *S*. *longiarticulata* (Nogueira, San Martín & Amaral, 2001) [21] **comb. nov.** (as *Grubeosyllis longiarticulata*) were originally described based on Brazilian specimens [21]. *Salvatoria neapolitana* (Goodrich, 1930) [23] also counts with some records among Brazilian material ([24], as *Brania subterranea* (Hartmann-Schröder, 1956) [25]. Other five species–*S*. *clavata* (Claparède, 1863) [26], *S*. *euritmica* (Sardá, 1984) [27], *S*. *heterocirra* (Rioja, 1941) [28], *S*. *limbata* (Claparède, 1868) [29] and *Salvatoria* cf. *nitidula* (Verrill, 1900) [18]–have also been found in the country, but recorded in unpublished works [20, 30, 31]. Out of all these species, only *S*. *limbata* does not have formal taxonomical accounts, neither vouchers of Brazilian material deposited in collections, so this record cannot be confirmed presently. Most of the species mentioned above were found only in Southeastern Brazil, specifically off the State of São Paulo. All of these records were from coastal, shallow water habitats.

In this paper we present a synopsis of the *Salvatoria* occurring in Brazilian waters, including specimens from the Northeastern coast, where the new species *Salvatoria nitiduloides* **n. sp.** was found; we also present the first account of *Salvatoria* from oceanic islands–important habitats relatively isolated from the continental shelf by large extension and with the deep sea in between–, where two new species, *S*. *ypsiloides* **n. sp.** and *S*. *marielleae* **n. sp.**, were found. Furthermore, material of *S*. *neapolitana*, *S*. cf. *nitidula* and *Salvatoria* sp. from Brazil are described and illustrated; also, we present the record and data of *S*. *breviarticulata* **comb. nov.**, *S*. *clavata*, *S*. *euritmica* and *S*. *longiarticulata* **comb. nov.** Finally, we provide a dichotomous identification key and a comparative table with biogeographical and taxonomic data on these species, with several characters considered informative for the systematics of *Salvatoria*.

## Material and methods

### Field permit and material analysed

Field Permits from Ministério do Meio Ambiente—MMA / Instituto Chico Mendes de Conservação da Biodiversidade—ICMBio and Sistema de Autorização e Informação em Biodiversidade—SISBIO were given to Rodolfo Leandro Nascimento Silva, João Miguel de Matos Nogueira and Karla Paresque (permits # 53344, 1947272 and 46541–1, respectively) to the sampling of organisms.

Type material and other examined specimens were deposited at Museu Nacional, Universidade Federal do Rio de Janeiro (MNRJP), Brazil, and Museu de Zoologia, Universidade de São Paulo (MZUSP), Brazil. Comparative material was examined from the United States National Museum, Smithsonian Institution (USNM), USA, and Museu de Zoologia, Universidade Estadual de Campinas (ZUEC-POL), Brazil (material from this museum is also quoted in the text under its former designation, MHN-BPO).

### Study area and sample processing

The specimens examined in this paper came from different projects: 1) ‘BIOTA/FAPESP/Benthic Marine Biodiversity in the State of São Paulo’ (‘*BIOTA’)*, focused on the fauna from off the Northern coast of São Paulo; 2) ‘Diversity of Polychaeta (Annelida) on rocky shores off the State of São Paulo, Southeastern Brazil’ (‘*BioPol-SP’*); 3) ‘Diversity of Polychaeta (Annelida) on sandstone reefs off Northeastern Brazil, States of Paraíba and Pernambuco’ (‘*BioPol-NE’*); 4) ‘ProTrindade Marine Invertebrate Project’ (‘*ProTrindade*’), focused on the fauna of the Trindade and Martin Vaz Archipelago, located about 1,140 km off the Southeastern coast of Brazil; 5) ‘Syllidae from Brazilian oceanic islands’; 6) Environmental Heterogeneity in the Campos Basin (‘*Habitats*’); (7) Environmental Characterization of the Espírito Santo Basin and the Northern Portion of the Campos Basin (‘*AMBES*’) and (8) Genetic connectivity along Brazilian coast: comparative phylogeography of two polychaete species with contrasting reproductive strategies.

The first three projects were conducted by the Laboratório de Poliquetologia (LaPol), Instituto de Biociências, Universidade de São Paulo (IB/USP). The fourth was conducted by the Laboratório de Carcinologia, Museu de Zoologia, Universidade de São Paulo (MZUSP). The fifth was conducted by the Laboratório de Polychaeta (LabPoly), Departamento de Zoologia, Universidade Federal do Rio de Janeiro (IB/UFRJ). The eighth was conducted by the Laboratório de Evolução Marinha (LEM), IB/USP. Projects ‘Habitats’ and ‘AMBES’, numbers 6 and 7, respectively, were focused on the soft bottom fauna from two ocean basins off Southeastern Brazil, which surveys were coordinated by the Brazilian energy company–PETROBRAS.

Examined material was extracted from algae, sponges, ascidians, scraped from similar substrates from rocky shores, sandstone and coral reefs, and from soft bottoms. Syllids were fixed immediately after collection in 4% formalin and preserved in 70–92% ethanol; some specimens were preserved directly in 100% ethanol.

Specimens were analysed under a Zeiss Stemi SV11 stereomicroscope and Zeiss Axio Lab A1 microscope. In addition, some specimens were examined using scanning electron microscopy (SEM). For the SEM, specimens were first dehydrated in a graded series of increasing concentrations of ethanol (up to 100%), critical point-dried, coated with ~30 nm of gold, and examined and photographed at the Laboratório de Imagem em Microscopia Óptica e Eletrônica (LABIM–UFRJ), Laboratório de Microscopia Eletrônica, MZUSP, and Laboratório de Microscopia Eletrônica, IB/USP. Line drawings were made from slide*-*mounted specimens with the aid of a drawing tube. The length of specimens was measured from the tip of palps to the tip of pygidium, excluding the anal cirri; width was measured at proventricular level, excluding parapodia.

**Fig 1:**
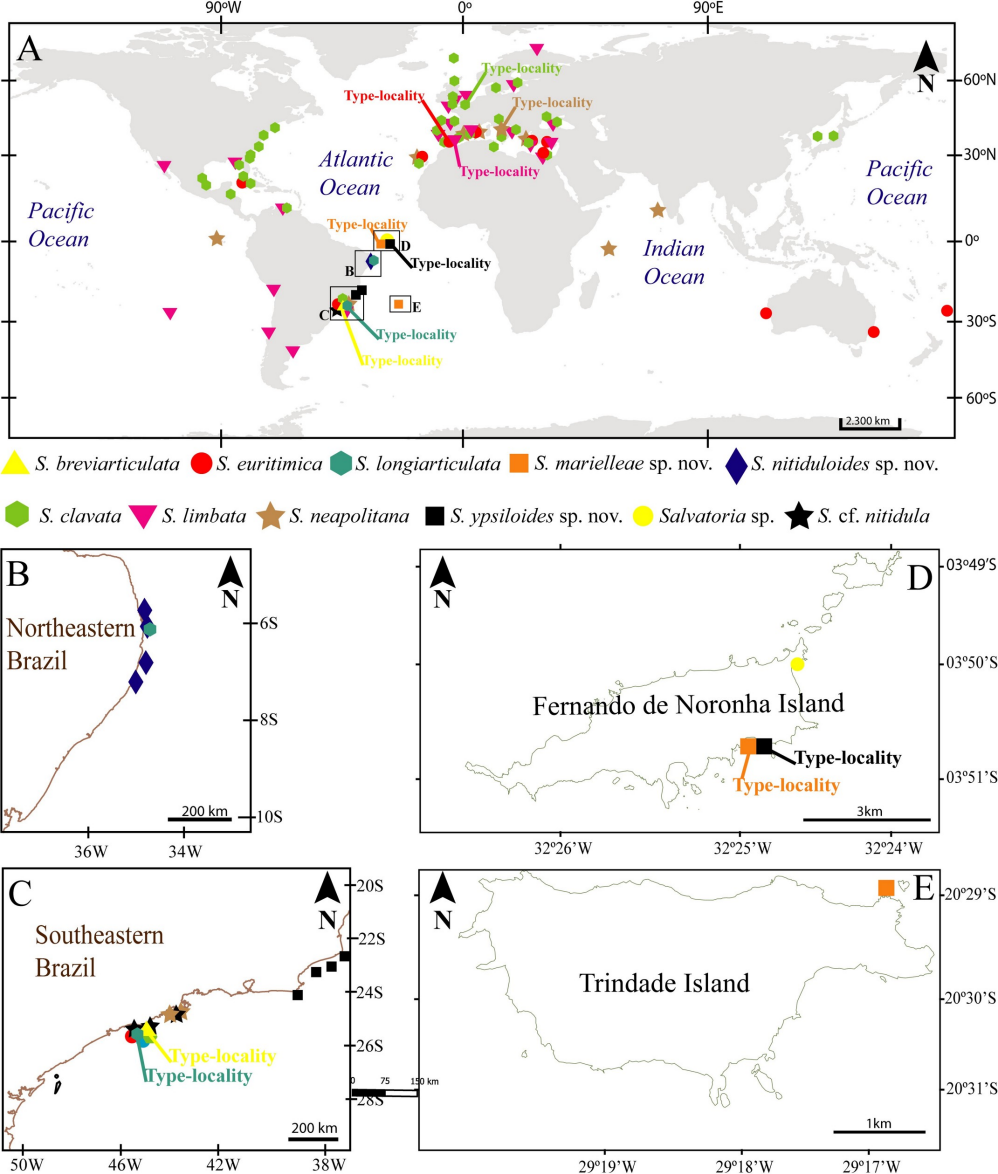
Distribution of the species of *Salvatoria* dealt with in the present paper. A, worldwide distribution records for the *Salvatoria* species registered in Brazil; B, C, species occurring on the Northeastern and Southeastern Brazilian coasts, respectively; D, E, species occurring on Fernando de Noronha and Trindade Islands, respectively.

Distribution records of the species presented herein (Fig 1) were extracted from published seminal papers with taxonomical approach [7, 17, 19–21], from data presented herein and also from the Global Biodiversity Information Facility [32], considering only the records based on ‘preserved specimens’ which are deposited in official collections.

### Nomenclatural acts

The electronic edition of this article conforms to the requirements of the amended International Code of Zoological Nomenclature, and hence the new names contained herein are available under that Code from the electronic edition of this article. This published work and the nomenclatural acts it contains have been registered in ZooBank, the online registration system for the ICZN. The ZooBank LSIDs (Life Science Identifiers) can be resolved and the associated information viewed through any standard web browser by appending the LSID to the prefix http://zoobank.org/. The LSID for this publication is: urn:lsid:zoobank.org:pub:B1D720CB-4B0F-4018-9B5F-134B67686970. The electronic edition of this work was published in a journal with an ISSN and has been archived and is available from the following digital repositories: PubMed Central, LOCKSS, ResearchGate and DigitalCSIC.

### Taxonomic account

Family Syllidae Grube, 1850 [1]

Subfamily Exogoninae Langerhans, 1879 [5]

Genus *Salvatoria* McIntosh, 1885 [14]

Type species: *Salvatoria kerguelensis* McIntosh, 1885 [14]

#### Diagnosis

Mid- to small-sized exogonines, body wall smooth, without papillae. Palps fused along most of their length by dorsal membrane, usually leaving short distal notch. Prostomium with three antennae, four eyes and sometimes two anterior eyespots. Two pairs of peristomial cirri. Dorsal cirri on chaetiger 2 present or absent. Antennae, peristomial and dorsal cirri throughout spindle-shaped to elongate, tapered. Ventral cirri digitiform, usually shorter than parapodial lobes. Parapodial glands absent. Compound chaetae as heterogomph falcigers, blades sub-bidentate to bidentate. Dorsal and ventral simple chaetae present. Aciculae usually subdistally sinuous and inflated, with long, filiform tip. Pharynx wide, with opening smooth or with marginal papillae in a few species; tooth rhomboidal to ovate, usually slightly away from anterior margin of pharynx. Proventricle relatively large, usually about as long as pharynx, with numerous slender muscle cell rows. Reproduction by epigamy, with dorsal brooding of eggs attached by capillary chaetae [17].

### Identification key for the *Salvatoria* species recorded in Brazilian waters

1a Palps more than twice as long as prostomium; falciger blades with faint dorsoventral gradation in length (11–8 μm long) throughout …….………*S*. *breviarticulata* **comb. nov.**

1b Palps just slightly longer, as long as or shorter than prostomium; falciger blades with conspicuous dorsoventral gradation in length, at least in one of body regions ………2

2a (1b) Dorsal simple chaetae distally bifid, “ypsiloid”, with unequal sizes; dorsal cirri distally hollow ……………………………….*S*. *ypsiloides* **n. sp.**

2b (1b) Dorsal simple chaetae different from above, uni- or bidentate; dorsal cirri not distally hollow ……………………………………………..……………………3

3a (2b) Dorsal simple chaetae unidentate…………… .… .… ..……………………..….4

3b (2b) Dorsal simple chaetae bidentate……………………………………….……..7

4a (3a) Antennae thick, conical, tapering distally; aciculae distally rounded, hollow; tooth at end of first ⅓ of pharynx ……… ..*S*. *neapolitana*

4b (3a) Antennae spindle shaped; aciculae distally tapering, oblique; tooth slightly away from anterior margin of pharynx…………………………………………………………5

5a (4b) Falciger blades unidentate, spines only on dorsalmost blade of each bundle…………*S*. *limbata*

5b (4b) Falciger blades bidentate and spinulated throughout… .… .…….6

6a (5b) Parapodial lobes smooth, without papillae; dorsal cirri subulated; dorsalmost falciger blades 33–34 μm long throughout; ventral simple chaetae bidentate………….*S*. *longiarticulata* **comb. nov.**

6b (5b) Parapodial lobes with 2–3 papillae; dorsal cirri distally tapered, with bases slightly thickened; dorsalmost falciger blades 20–22 μm long throughout; ventral simple chaetae unidentate………..*Salvatoria* sp.

7a (3b) Parapodial lobes distally papillated …..……………..8

7b (3b) Parapodial lobes smooth, without papillae…………………..……………………9

8a (7a) Palps dorsally united for most of their length, only leaving a distal notch; falciger blades with distal tooth larger throughout, oblique space between teeth; dorsal simple chaetae starting from first chaetiger…………..*S*. *clavata*

8b (7a) Palps dorsally united for up to half their length; falciger blades with teeth similar in size, rounded space between teeth; dorsal simple chaetae starting from midbody.……..*S*. *euritmica*

9a (7b) Antennae typically spindle shaped, all similar in size; dorsal cirri digitiform, tapered, midbody dorsal cirri longer than corresponding chaetiger width; falciger blades with teeth similar in size throughout, with rounded space between teeth…….…*S*. *marielleae* **n. sp.**

9b (7b) Antennae spindle shaped to subulated, median antenna longer; dorsal cirri spindle shaped or subulated, midbody dorsal cirri shorter than corresponding chaetiger width; falciger blades with teeth of unequal sizes at least in some region of body……10

10a (9b) Median antenna twice as long as lateral antennae, inserted posteriorly on prostomium, between posterior eyes; falciger blades 18–8 μm long throughout; proventricle with up to 28 muscle cell rows…….*S*. *heterocirra*

10b (9b) Median antenna slightly longer than lateral antennae, inserted more anteriorly, between anterior eyes; falciger blades longer than above; proventricle with up to 21 muscle cell rows…………….11

11a (10b) Dorsal cirri of chaetiger 1 longer than others; falciger blades with teeth about same size only on posterior body; ventral simple chaetae smooth, bidentate, teeth about same size …..*S*. cf. *nitidula*

11b (10b) Dorsal cirri of chaetigers 2 and 3 shorter, those of chaetigers 4 and 5 as long as cirri of chaetiger 1; falciger blades with distal tooth larger than subdistal one throughout; ventral simple chaetae spinulated, bidentate, distal tooth slightly larger than subdistal one…………*S*. *nitiduloides* **n. sp.**

#### Remarks

Some species in the key were not examined for the present article, characters based on the literature: *S*. *breviarticulata*, *S*. *heterocirra* and *S*. *longiarticulata* from [20]; *S*. *limbata*, *S*. *clavata* and *S*. *euritmica* from [7] and [20].

***Salvatoria longiarticulata* (Nogueira, San Martín & Amaral, 2001) [21] comb. nov.**

Table 1

**Table 1:** Taxonomic and biogeographic data on the species of *Salvatoria* recorded for Brazilian waters, with reference to voucher material.

	Original description	Brazilian material	Source of Records	Body length x width (mm) / n° of chaetigers	Palps	Antennae
*S*. *breviarticulata* comb. nov.	[21]	MHN-BPO 67/0. MHN-BPO 67/1–3	[9,20,21]	1.9 x 0.2 / 26	More than twice as long as prostomium, united up to mid-length	Spindle shaped
*S*. *clavata*	[26]	MHN-BPO JN 14/1–4	[9,20]	3.5 x 0.35 / 31	As long as prostomium, almost completely united, leaving a distal notch	Spindle shaped
*S*. *euritmica*	[27]	MHN-BPO JN 16/1–3	[9,20]	4 x 0.35 / 29	As long as prostomium, united up to mid-length	Spindle shaped
*S*. *heterocirra*	[28]	MHN-BPO JN 15/1–25	[9, 20, 24]	? x 0.9 /??	Longer than prostomium, almost completely united, leaving a distal notch	Spindle shaped
*S*. *limbata*	[29]	Without material deposited	[9]. Doubtful record	2 x 0.15 / 30	Shorter than prostomium, almost completely united, leaving a distal notch	Spindle shaped
*S*. *longiarticulata* comb. nov.	[21]	MHN-BPO 68/0. MHN-BPO 68/1–5.	[9, 20, 21, 30], this paper	2.47 x 0.17 / 28	Shorter than prostomium, almost completely united, leaving a distal notch	Spindle shaped
*S*. *marielleae* n. sp.	This paper	MNRJP and MZUSP (see below)	This paper	2.3 x 0.18 / 22	As long as prostomium, almost completely united, leaving a distal notch	Spindle shaped
*S*. *neapolitana*	[23]	MZUSP (see below)	[30], this paper	1.8 x 0.15 / 23	Longer than prostomium, almost completely united, leaving a distal notch	Conical, elongated, thick, distally pointed
*S*. cf. *nitidula*	[18]	MZUSP (see below)	[30], this paper	2.37 x 0.18 / 31	As long as prostomium, almost completely united, leaving a distal notch	Spindle shaped to subulated
*S*. *nitiduloides* n. sp.	This paper	MZUSP (see below)	This paper	2.2 x 0.2 / 29	As long as prostomium, almost completely united, leaving a distal notch	Spindle shaped to subulated
*S*. *ypsiloides* n. sp.	This paper	MNRJP and MZUSP (see below)	This paper	2.2 x 0.12 / 32	Shorter than prostomium, almost completely united, leaving a distal notch	Spindle shaped to digitiform
*Salvatoria* sp.	This paper	MZUSP (see below)	This paper	1.3 x 0.14 / 20	Shorter than prostomium, completely united	Spindle shaped
	**Dorsal cirri**		**Parapodia**	**Number of falcigers (anterior / posterior)**	**Falciger blades morphology**	**Falciger blades length (anterior / posterior blades (μm))**
*S*. *breviarticulata* comb. nov.	Same shape as antennae		Smooth	4 throughout	Bidentate, distal tooth larger throughout, oblique space inbetween	11–8 / 10 from midbody
*S*. *clavata*	Same shape as antennae		2–3 papillae	10 / 4–7	Bidentate, distal tooth larger throughout, oblique space inbetween	19–12 / 12–11
*S*. *euritmica*	Same shape as antennae		2–3 papillae	12 / 5–6	Bidentate, teeth similar in size, rounded space inbetween	31–20 / 12–9
*S*. *heterocirra*	Different from antennae, spindle shaped to subulate		Smooth	??	Bidentate, distal teeth similar in size throughout, oblique space inbetween	18–8 throughout
*S*. *limbata*	Different from antennae, spindle shaped to subulate		2–3 papillae	10–9 / 6–7	Unidentate	24–14 / —
*S*. *longiarticulata* comb. nov.	Different from antennae, spindle shaped to subulated		Smooth	6 throughout	Bidentate, distal tooth longer, subdistal tooth very slender; oblique space inbetween	34–18 throughout
*S*. *marielleae* n. sp.	Different from antennae, distally tapered, with bases slightly thickened		Smooth	7–5 throughout	Bidentate, teeth similar in size; rounded space inbetween	21‒9 / 20‒10
*S*. *neapolitana*	Same shape as antennae		1 papilla	10 / 2–5	Sub-bidentate, subdistal tooth thin, spine-like; oblique space inbetween	19–9 throughout
*S*. cf. *nitidula*	Same shape as antennae		Smooth	7–6 throughout	Bidentate, distal tooth slightly larger on anterior and midbody, teeth about same size on posterior body; oblique space inbetween	30–10 / 25–10
*S*. *nitiduloides* n. sp.	Same shape as antennae		Smooth	10–7 / 3–4	Bidentate, distal tooth slightly larger throughout; oblique space inbetween	22–8 / 24–11
*S*. *ypsiloides* n. sp.	Different from antennae, elongate, digitiform, sometimes slightly swollen distally hollow		Smooth	7–5 throughout	Bidentate, distal tooth slightly shorter on anterior body; teeth about same size on mid- and posterior body; wide angle inbetween	18‒7.4 / 19‒8
*Salvatoria* sp.	Different from antennae, distally tapered, with bases slightly thickened		2–3 papillae	7‒6 / 3	Bidentate, distal tooth slightly larger; oblique space inbetween	22‒7 / 20‒6
	**Dorsal simple chaeta**		**Ventral simple chaeta**	**Number of pharynx segments / tooth position**	**Number of proventricle segments / muscle cell rows**	**Habitat**	**Distribution**
*S*. *breviarticulata* comb. nov.	Present from midbody; bidentate		Only on posterior parapodia, sigmoid; slightly bidentate	4 / at anterior margin	4 / 23	Coastal, subtidal zones, 4–73 m deep. Associated with *M*. *hispida* (Verrill, 1868) coral heads	Only known from type locality. Atlantic Ocean: Brazil (**São Paulo**).
*S*. *clavata*	Present from first chaetiger; bidentate		Only on posterior parapodia; straight, bidentate	3–4 / at first ⅓	3–4 / 20–23	Coastal and oceanic, intertidal to 46 m deep. Associated with algae, corals, bryozoans, calcareous algae, but also found in soft sediments	Atlantic Ocean: Norwegian Sea, **Normandy**, Mediterranean Sea, USA, Cuba, Brazil (São Paulo). Pacific Ocean: Sea of Japan.
*S*. *euritmica*	Present from midbody; bidentate		Only on posterior parapodia; sigmoid, bidentate	3–4 / slightly away from anterior margin	~4 / 15–20	Coastal and oceanic, intertidal to 44 m deep. Associated with corals, algae, and seagrass, but also found in soft sediments	Atlantic Ocean: Southern Mediterranean Sea (**Gibraltar**), Caribbean Sea, Brazil (São Paulo). Pacific Ocean: Australia.
*S*. *heterocirra*	Bidentate		Slightly sigmoid, bidentate.	~4 / slightly away from anterior margin	2–2.5 / 28	Coastal and oceanic, intertidal to 20 m deep. Associated with corals, but also found in soft sediments	Atlantic Ocean: Gulf of Mexico, Brazil (São Paulo). Pacific Ocean: **Mexico**, Costa Rica, Panamá.
*S*. *limbata*	Present from first chaetiger; unidentate		Only on posterior parapodia; curved, unidentate	~3 / slightly away from anterior margin	~3 / 16–18	Coastal and oceanic, intertidal to 144 m deep. Associated with algae, corals, sponges, but also found in soft sediments	Atlantic Ocean: Mediterranean Sea (**France**), Caribbean Sea, Brazil (São Paulo), Argentina. Pacific Ocean: Mexico, Chile, Australia.
*S*. *longiarticulata* comb. nov.	Present from anterior body; unidentate		Only on posterior body; sigmoid, bidentate	3–4 / slightly away from anterior margin	3–4 / 24	Coastal, intertidal to 7 m deep. On rocky shores and associated with *M*. *hispida* coral heads	Atlantic Ocean: Brazil (Paraíba, Pernambuco, **São Paulo**)
	**Dorsal simple chaeta**	**Ventral simple chaeta**	**Number of pharynx segments / tooth position**	**Number of proventricle segments / muscle cell rows**	**Habitat**	**Distribution**
*S*. *marielleae* n. sp.	Present from proventricle level; bidentate	Only on posterior parapodia; sigmoid, bidentate	3 / at anterior margin	~2.5 / 18‒19	Oceanic, intertidal to 12 m deep. Associated with red algae	Atlantic Ocean: Brazil (Pernambuco–**Fernando de Noronha island)**
*S*. *neapolitana*	Present from first chaetiger; unidentate	Only on posterior parapodia; slightly curved, unidentate	3 / at first ⅓	3.5 / 20	Coastal and oceanic, intertidal to 19 m deep. Associated with algae, but also found in soft sediments	Atlantic Ocean: Mediterranean Sea, France, Spain, **Italy (Gulf of Naples**), Canary Islands, Venezuela, Caribbean Sea, Brazil (São Paulo). Pacific Ocean: Australia
*S*. cf. *nitidula*	Present from first chaetiger; bidentate distal tooth slightly longer	Only on posterior parapodia; sigmoid, bidentate, smooth	3.5–4 / slightly away from anterior margin	3–4.5 / 16–20	Coastal, intertidal. Associated with algae and similar substrates on rocky shores	Records of original description–Atlantic Ocean: **Bermuda;** Caribbean Sea (Gulf of Mexico, Cuba). Current records: SE Brazil (São Paulo)
*S*. *nitiduloides* n. sp.	Present from anterior body; bidentate, teeth similar in size	Only on posterior parapodia; sigmoid, bidentate, spinulated	3–4 / slightly away from anterior margin	2–3 / 19–22	Coastal, intertidal. Associated with algae and similar substrates on rocky shores	Atlantic Ocean: Brazil (Paraíba, Pernambuco**–Ilha de Itamaracá**)
*S*. *ypsiloides* n. sp.	Present from first chaetiger; bifid, “ypsiloid” in appearance	From midbody parapodia; sigmoid, bidentate	4–5 / slightly away from anterior margin	4.5‒5.5 / 26‒29	Coastal and oceanic, subtidal to 970 m deep. Associated with red algae, soft sediments	Atlantic Ocean: Brazil (Pernambuco–**Fernando de Noronha island**, Espírito Santo, Rio de Janeiro)
*Salvatoria* sp.	Present from chaetiger 2; unidentate	Only on posterior body; sigmoid, unidentate	3.5 / slightly away from anterior margin	~3 / 15	Oceanic, intertidal to 1 m deep. Associated with sponge *Plakortis insularis*	Atlantic Ocean: Brazil (Pernambuco–Fernando de Noronha)

Type localities in bold. Data of *S*. *breviarticulata* and *S*. *longiarticulata* from [21], *S*. *limbata*, *S*. *clavata* and *S*. *euritmica* from [7] and [20].

*Grubeosyllis longiarticulata* Nogueira, San Martín & Amaral, 2001: 1788–1791, Fig 5 [21].

#### Material examined

Project *BioPol-NE*—State of Paraíba, João Pessoa, Praia do Cabo Branco (7°08’S 34°47’W), intertidal: 20 specimens, coll. 02 February 2010.

#### Remarks

The material analysed herein matches the original description provided by [21] from São Paulo, except for details on the teeth of falciger blades and a slight variation on the number of muscle cells rows in the proventricle. Specimens from Paraíba have both teeth shorter and directed forwards, and proventricle with ca. 17 rows of muscle cells, while material from São Paulo has falciger blades with longer teeth directed slightly upwards, and 24 rows of proventricular muscle cells.

#### Type locality

Ilha dos Alcatrazes, Santos, São Paulo, Brazil (SW Atlantic).

#### Distribution

Southwestern Atlantic, Brazil (states of Paraíba and São Paulo). First record for this species in Northeastern Brazil and also after original description (Fig 1A–1C).

***Salvatoria* cf. *nitidula* (Verrill, 1900) [18] comb. nov.**

Fig 2, Table 1.

**Fig 2:**
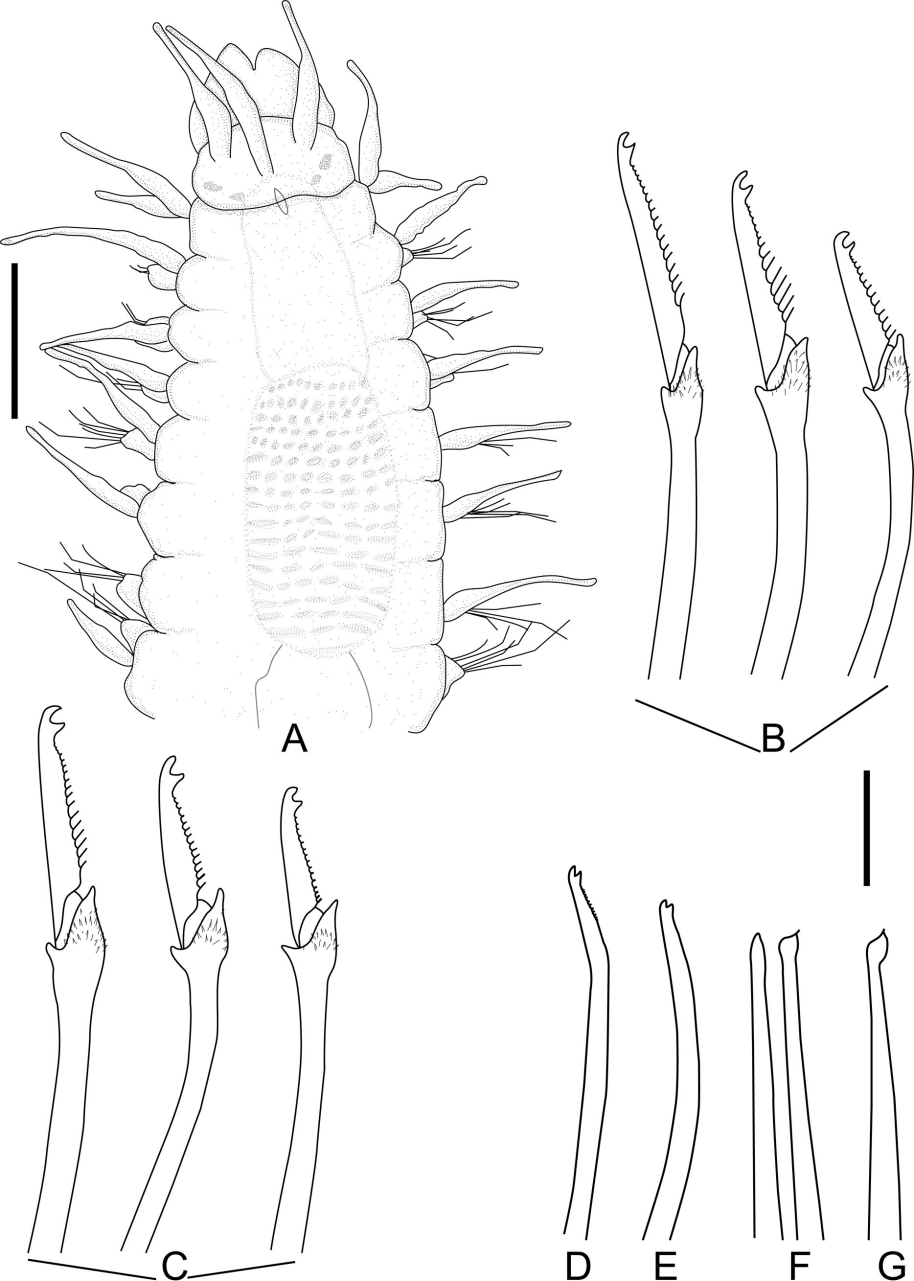
*Salvatoria* cf. *nitidula*. A, anterior body, dorsal view; B, falcigers, anterior and midbody parapodia; C, falcigers, posterior parapodium; D, dorsal simple chaeta; E, ventral simple chaeta; F, G, aciculae, anterior and posterior body, respectively. Scale bars: A, 0.11 mm; B–G, 10 μm.

*Salvatoria nitidula*. Fukuda, 2010 [30]: 183–187, Fig 54.

#### Material examined

Project ‘*BIOTA’*—State of São Paulo, Ubatuba, Praia de Picinguaba (23°22’31’’S 44°50’21’’W), on rocky shore: 2 specimens (MZUSP 4266), coll. 9 May 2001; 8 specimens (MZUSP 4267), coll. 10 May 2001; 1 specimen, coll. 11 October 2001; 11 specimens (MZUSP 4268), coll. 17 October 2001; on *Sargassum* sp.: 124 specimens (MZUSP 4269), coll. 18 October 2001; 2 specimens (date unknow); on phytal: 1 specimen (MZUSP 4270), coll. 8 October 2001. Caraguatatuba–Praia Martim de Sá (23°37’34’’S 45°22’31’’W), on rocky shore: 9 specimens (MZUSP 4264), coll. 19 September 2001; 3 specimens (MZUSP 4265), coll.21 September 2001; 2 specimens (date unknow); on *Sargassum* sp.: 1 specimen, coll. 27 September 2001; 2 specimens, coll. 16 March 2001. São Sebastião–Praia da Baleia (23°46’48’’S 45°39’51’’W), on rocky shore: 2 specimens (MZUSP 4263), coll. 13 December 2001; Praia de Toque-Toque Grande (23°50’12’’S 45°30’40’’W), on rocky shore: 1 specimen, coll. 10 April 2001. Project ‘*BioPol*-*SP’*—Ubatuba–Praia do Perequê-Mirim (23°29’20’’S 45°06’25’’W): 2 specimens, coll. 5 January 2003. São Sebastião–Praia do Araçá (23°48’54’’S 45°24’24’’W): 1 specimen, coll. 17 April 2003; 3 specimens, coll. 15 July 2003; 6 specimens, coll. 25 September 2003; 4 specimens (MZUSP 4262), coll. 20 July 2005. Santos–Ilha das Palmas (24°00’34’’S 46°19’25’’W): 2 specimens, coll. 5 October 2005.

#### Description

Small-sized body; longest specimen examined 2.37 mm long and 0.18 mm wide, with 31 chaetigers (Table 1). Palps subtriangular, almost totally fused, leaving a distal notch (Fig 2A). Prostomium subrectangular, as long as palps (Fig 2A), with two pairs of eyes in open trapezoidal arrangement (Fig 2A), anterior eyespots usually present; antennae spindle-shaped to slightly subulated, median antenna inserted between eyes, slightly longer than prostomium and palps together, lateral antennae slightly shorter than median antenna, inserted anteriorly on prostomium, slightly away from margin, reaching beyond tips of palps (Fig 2A). Peristomium as long as anterior body chaetigers; peristomial cirri with same shape as antennae; dorsal peristomial cirri slightly shorter than median antenna; ventral peristomial cirri about half length of dorsal ones (Fig 2A). Dorsal cirri with same shape as antennae; dorsal cirri of chaetiger 1 slightly longer than median antenna, subsequent dorsal cirri short, similar in length to lateral antennae (Fig 2A). Ventral cirri digitiform, inserted at bases of parapodial lobes. Parapodial lobes smooth, distally bilobate. Anterior and midbody parapodia with 6 falcigers each throughout. Shafts of falcigers slightly spinulated, subdistally inflated and distally tapered (Fig 2B and 2C). Blades of falcigers spinulated, bidentate, distal tooth slightly larger than subdistal one on anterior and midbody parapodia (Fig 2B), teeth about same size on posterior parapodia (Fig 2C); blades of falcigers with dorso-ventral gradation in length (Fig 2B and 2C), 30–10 μm long on anterior and midbody parapodia, 25–10 μm long on posterior parapodia (Table 1). Dorsal simple chaetae present from chaetiger 1, slightly thinner than shafts of falcigers of same fascicle, with short subdistal spines, distally bidentate, distal tooth slightly longer (Fig 2D); ventral simple chaetae only present on posteriormost parapodia, sigmoid, smooth, bidentate, teeth similar in size (Fig 2E). Anterior parapodia with two aciculae each, one of which thin, straight, distally tapered, with acute tip sometimes protruding from parapodial lobe, other acicula subdistally inflated and slightly twisted, with acute tip (Fig 2F); single acicula per parapodium from midbody, subdistally inflated and slightly twisted, with long and thin, needle-like tip (Fig 2G). Semicircular pygidium with paired anal cirri, similar in shape to dorsal cirri. Pharynx through 3.5–4 segments (Fig 2A); tooth relatively long, rhomboidal to ovate, slightly away from anterior margin of pharynx; proventricle extending for 3–4.5 chaetigers, with 16–20 rows of muscle cells (Table 1).

#### Remarks

The species *Salvatoria nitidula* was described from Bermuda, in the North Atlantic American coast, so the presence of animals belonging to this species along the Brazilian coast would not be totally surprising. However, the original description of the species is very brief and does not mention important characters, such as details of pharyngeal structures and compound chaetae. San Martín [19] reported *S*. *nitidula* (as *Grubeosyllis nitidula*), describing animals similar to those herein analysed, from Cuba, Bermuda, and the Gulf of Mexico, however, with considerable doubts due to the lack of information in the original description. One noticeable difference is related to the length of dorsal and ventral peristomial cirri: about this character, in the original description Verrill [18] describes “*Tentacular cirri two on each side*, *of about the same length*”, while both our specimens and those analysed by [19] show ventral peristomial cirri with about half length of dorsal ones. Nonetheless, herein we follow [19] in also assigning this species with caveats, until we can examine the syntypes or a redescription based on Bermudan specimens is provided.

The Mediterranean *Salvatoria vieitezi* (San Martín, 1984) [16] shares with *S*. cf. *nitidula* the size of the proventricle, the spinulation pattern of falciger blades and the morphology of aciculae, dorsal and ventral simple chaetae. However, *S*. *vieitezi* differs from *S*. cf. *nitidula* by having only one acicula per parapodium throughout, subdistally curved, with acute tip; blades of dorsalmost falcigers up to 20 μm long, with long spines directed upwards, more conspicuous at mid-length of blades; intermediate and ventralmost falcigers with blades with straight spines slightly shorter than in *S*. cf. *nitidula*, 13 μm and 10 μm long, respectively. *S*. *vieitezi* can be also differentiated by its pigmentation pattern, with dark-red transverse stripes on the dorsum of each segment, more evident in live animals, but often preserved in fixed material.

Another species similar to *Salvatoria* cf. *nitidula* by sharing the overall morphology of antennae and peristomial and dorsal cirri throughout is *Salvatoria heterocirra* (Rioja, 1941) [28], described from the Pacific coast of Mexico. However, *S*. *heterocirra* differs from *S*. cf. *nitidula* by having only one acicula per parapodium throughout, distally rounded, hollow; falciger blades with short spinulation, distally bidentate, with subdistal tooth of even sizes throughout, without variation; and proventricle slightly smaller, through 2–2.5 segments [28]. On the other hand, *S*. cf. *nitidula* has two aciculae per parapodium on anterior body, falciger blades with subdistal tooth varying in size along the body and proventricle extending through ~3–4.5 segments.

*Salvatoria heterocirra* was identified among Brazilian material by [24], based on body size, number of segments and of eyes, shape of palps, antennae and peristomial cirri, length of dorsal cirri of the chaetiger 1 and length and number of muscle cell rows of the proventricle; on the occasion, the author considered the pharyngeal size as the only difference between his specimens and the original description of *S*. *heterocirra*. Nonetheless, the set of characters used by [24] to justify this identification is shared by several species within the genus. Moreover, comparing the description presented by [24] with the original description of the species [28], a discrepancy in the shape of falciger blades was noticed. Although this character was not described in detail, the drawings by [28] illustrate the falciger blades of *S*. *heterocirra* as having small size variation, short spinulation and approximately equal sized teeth. In addition, [28] described the species as having only 1 acicula per parapodium, differing from [24], which follows the pattern described herein for *S*. cf. *nitidula*. [24] informs that type material of *S*. *heterocirra* was not analysed for that study, but also makes no reference to these differences in his discussion. *S*. *heterocirra sensu* [24] was also later identified in the Brazilian coast, with restrictions, by [20] (as *Grubeosyllis* cf. *heterocirra*), perhaps in both cases being the species identified herein as *S*. cf. *nitidula*. To proper address this question and solve this issue it is necessary to reanalyses the material studied by [20, 24], but this has not been done for the present paper.

#### Distribution

South Atlantic, Brazil (State of São Paulo) (Fig 1A and 1C). Current distribution of *S*. *nitidula*—Atlantic Ocean: Bermuda (type locality), Caribbean Sea, Gulf of Mexico, Cuba [19].

***Salvatoria nitiduloides* n. sp.**

urn:lsid:zoobank.org:act:781214DA-DD13-42BE-9FE2-09113289A79F

Figs 3–5, Table 1.

**Fig 3:**
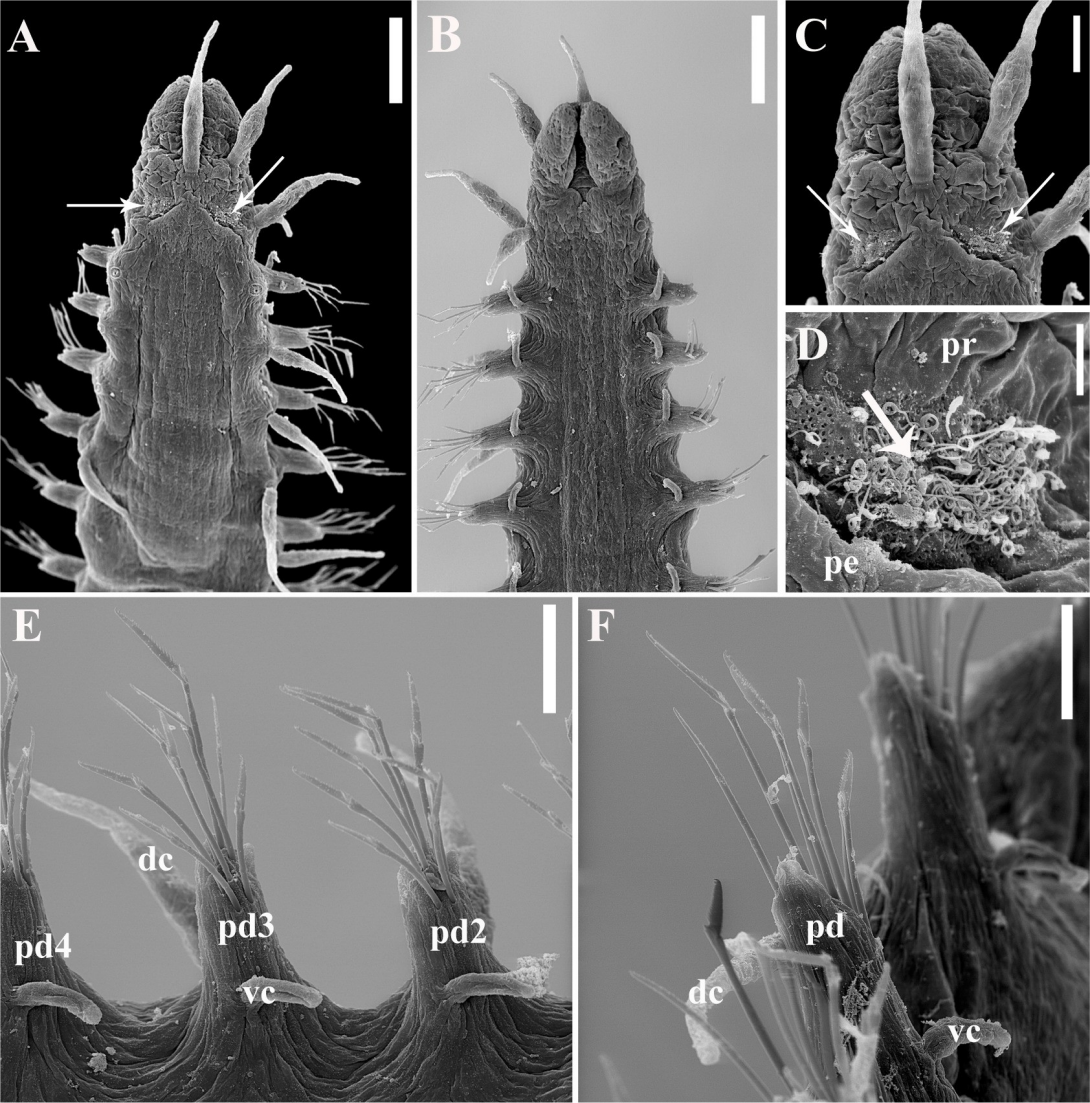
*Salvatoria nitiduloides* n. sp., SEM. (A, B). Anterior body, dorsal and ventral views, respectively; (C). Close up view of the anterior end, dorsal view; (D). Detail of ciliated nuchal organ; (E). Parapodia 2–4, ventral view; (F). Posterior parapodia, ventro-lateral view. Arrows pointing to ciliated nuchal organs; **dc**–dorsal cirrus; **pd**–parapodial lobe; **pd2, pd3, pd4** –parapodial lobes of chaetigers 2, 3 and 4 respectively; **pe**–peristomium; **pr**–prostomium; **vc**–ventral cirrus. Scale bars: A–B, 50 μm; C, E–F, 20 μm; D, 5 μm.

**Fig 4:**
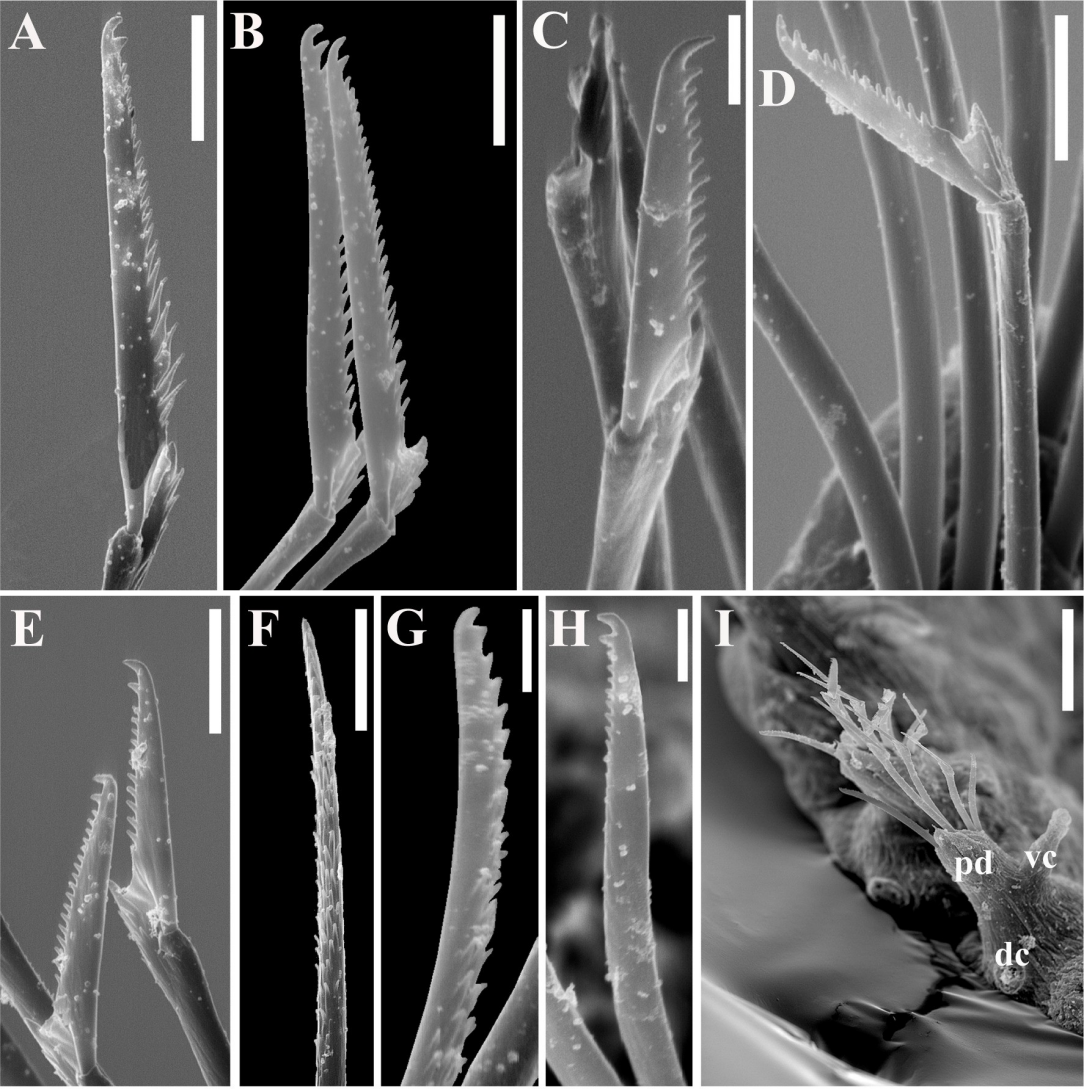
*Salvatoria nitiduloides* n. sp., SEM. (A, B). Dorsalmost falcigers, anterior parapodium; (C). Ventralmost falcigers, parapodium 2; (D). Ventralmost falciger, midbody parapodium; (E). Ventralmost falcigers, posterior parapodium; (F–G). Dorsal simple chaetae, posterior parapodium; (H). Ventral simple chaeta, posterior parapodium; (I). Posterior parapodia, frontal view. **pd**–parapodial lobe; **vc**–ventral cirrus; **dc**–scar of dorsal cirrus insertion (cirrus absent). Scale bars: A–B, D–F, 5 μm; C, G–I, 2 μm.

**Fig 5:**
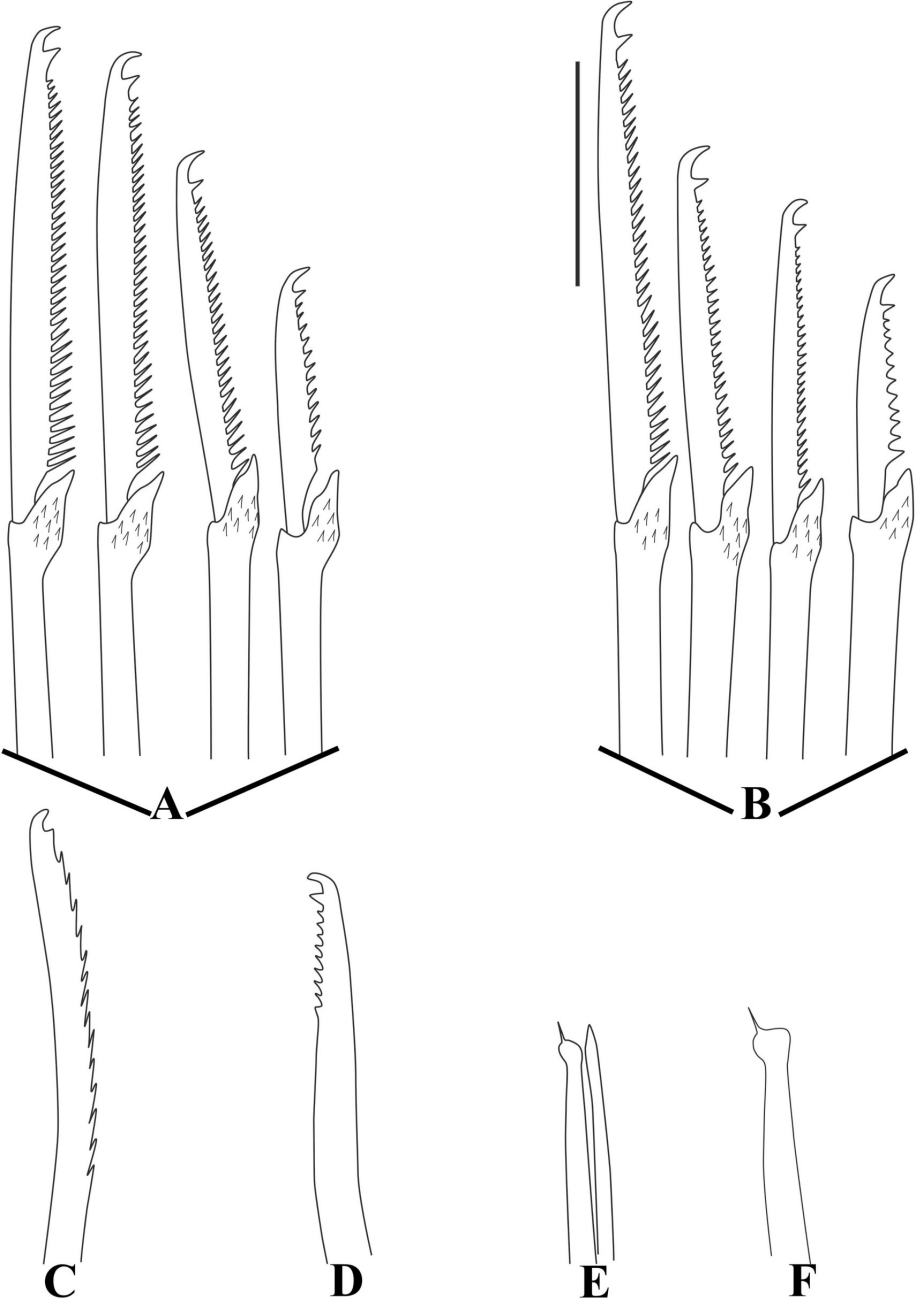
*Salvatoria nitiduloides* n. sp. (A, B). Falcigers, anterior and posterior parapodia, respectively; (C). Dorsal simple chaeta, posterior parapodium; (D). Ventral simple chaeta posterior parapodium; E, F, aciculae, anterior and posterior parapodia, respectively. Scale bar: 10 μm.

*Salvatoria* cf. *nitidula*. Paresque, 2014 [31]: 310–313, Figs 3–6.

**Fig 6:**
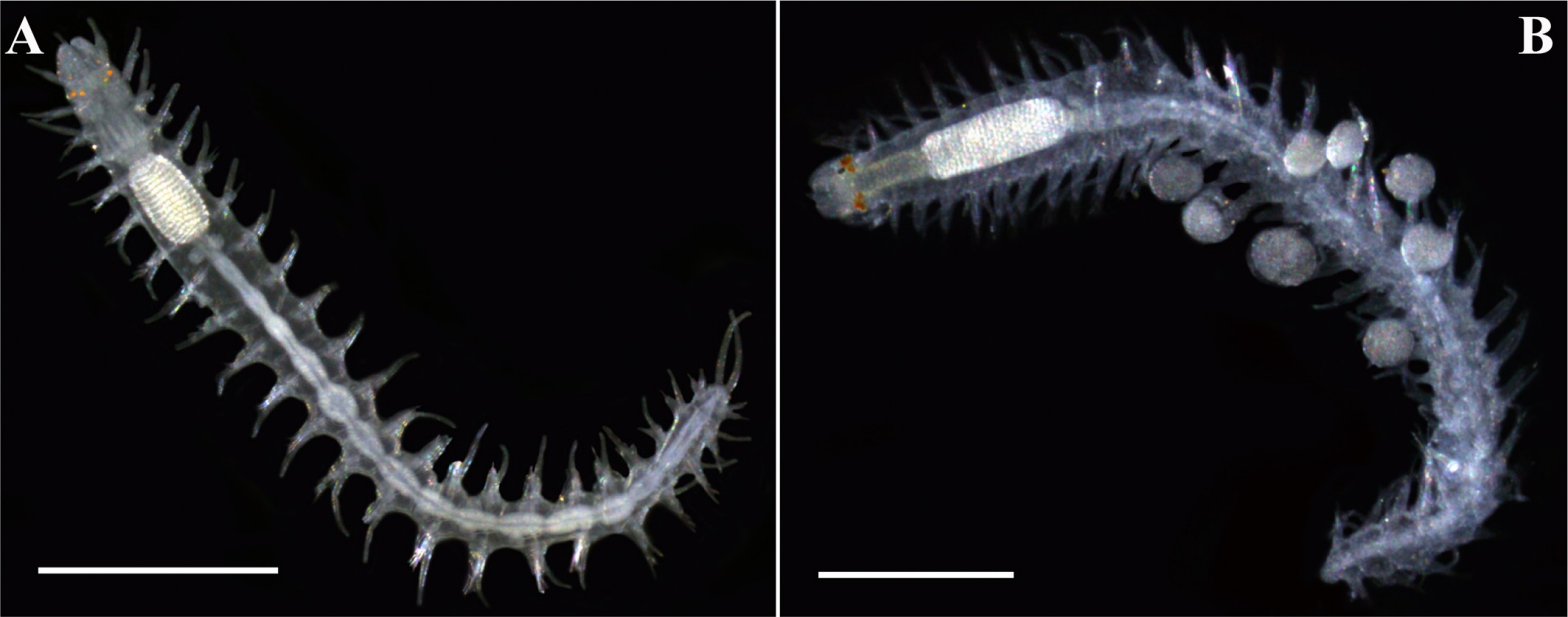
Photographs of the holotypes. (A). *Salvatoria marielleae* **n. sp.**, entire body, dorsal view; (B). *Salvatoria ypsiloides* **n. sp.** brooding eggs dorsally, entire body, dorsal view. Scale bars: A, 0.5 mm; B, 0.3 mm.

#### Type series

*Holotype*: *Project ‘BioPol-NE’*—State of Pernambuco, Ilha de Itamaracá, recifes de Itamaracá (07°43.9’S 34°49.2’W), 1 m deep: (MZUSP 4824), coll. 15 December 2012. *Paratypes*:—State of Paraíba, Praia do Coqueirinho (07°18’S 34°47’W), Paratype 1 (MZUSP 4825), coll. 28 August 2011; Paratype 2 (MZUSP 4826), Paratype 3 (MZUSP 4827), coll. 01 September 2011.—State of Pernambuco, Tamandaré, Praia dos Carneiros (08°42.8’S 35°4.9’W), intertidal: Paratype 4 (MZUSP 4828), coll. 22 June 2013.

#### Material examined

Project *‘BioPol-NE’*—State of Paraíba, Mataraca, Barra de Camaratuba (06°36’S 34°57’W), intertidal: 15 specimens, coll. 12 August 2010; Baía da Traição, Praia do Farol (06°41’S 34°55’W), intertidal: 6 specimens, coll. 09 August 2010; Rio Tinto, Barra de Mamanguape (06°45’S 34°55’W), intertidal: 24 specimens, coll. 11 August 2010; Cabedelo, Píer de Cabedelo (06°58’S 34°50’W), intertidal: 2 specimens, coll. 12 February 2009; João Pessoa, Praia do Cabo Branco (07°08’S 34°47’W), intertidal: 1 specimen, coll. 09 February 2009; 23 specimens, coll. 02 February 2010; Recife do Picãozinho (07°4.2’S 34°49.3’W), 1 m deep: 2 specimens, coll. 15 September 2012; Conde, Praia de Jacumã (07°14’S 34°47’W), intertidal: 3 specimens, coll. 29 January 2010; Praia de Tabatinga (07°19’S 34°47’W), intertidal: 15 specimens; coll. 17 September 2012; Praia do Coqueirinho (07°18’S 34°47’W), 12 specimens, coll. 28 August 2011; Praia de Tambaba (07°21’S 34°47’W), 1 specimen, coll. 30 August 2011. State of Pernambuco, Goiana, Pontas de Pedra (07°37’S 34°48’W), 4 specimens, coll. 13 December 2012; Ilha de Itamaracá, recifes de Itamaracá (07°43.9’S 34°49.2’W), 1 m deep: 13 specimens, coll. 15 December 2012; Ponta do Jaguaribe (07°44’S 34°49’W), intertidal: 5 specimens, coll. 11 December 2012; Tamandaré, Praia dos Carneiros (08°42.8’S 35°4.9’W), intertidal: 1 specimen, coll. 22 July 2013; Sirinhaém, Barra do Sirinhaém (08°36.7’S 35°2.4’W): 8 specimens, coll. 23 July 2013.

#### Description

Small-sized body; longest specimen examined 2.2 mm long, 0.20 mm wide, with 29 chaetigers. Palps triangular, distally rounded, almost totally fused, leaving a distal notch (Fig 3A and 3C). Prostomium rectangular, shorter than palps (Fig 3A and 3C), with two pairs of eyes in open trapezoidal arrangement, anterior eyespots usually present; antennae spindle-shaped to slightly subulated, median antenna inserted between eyes, longer than prostomium and palps together, lateral antennae inserted on anterior margin of prostomium, reaching beyond tips of palps (Fig 3A). Ciliated nuchal organs between prostomium and peristomium (Fig 3A, 3C and 3D). Peristomium as long as anterior body chaetigers; peristomial cirri with same shape as antennae; dorsal peristomial cirri as long as median antenna; ventral peristomial cirri about half length of dorsal ones (Fig 3A and 3B). Dorsal cirri of chaetiger 1 slightly longer than median antenna, dorsal cirri of chaetigers 2 and 3 shorter (Fig 3A), dorsal cirri of chaetigers 4 and 5 as long as dorsal cirri of chaetiger 1 or slightly shorter (Fig 3A); following dorsal cirri irregularly alternating long, as long as body width at corresponding chaetiger or slightly shorter, and short, shorter than body width at corresponding chaetiger. Ventral cirri digitiform, inserted at bases of parapodial lobes, not reaching their tips (Figs 3B, 3E, 3F and 4I). Parapodial lobes, smooth, distally bilobate (Fig 3A and 3E). Anterior and midbody parapodia with 7–10 falcigers each; posterior parapodia with 3–4 each. Shafts of falcigers slightly spinulated and inflated subdistally, tapering distally (Figs 4A–4E, 5A and 5B). Blades of falcigers spinulated, bidentate, distal tooth larger than subdistal one throughout (Figs 4A–4E, 5A and 5B); within each fascicle, blades of dorsalmost falcigers with longer spines than remaining falcigers (Figs 4A–4E, 5A and 5B); blades of falcigers with dorso-ventral gradation in length, 22–8 μm, 25–12 μm and 24–11 μm long on anterior, mid- and posterior body parapodia, respectively. Dorsal simple chaetae present from anterior parapodia, slightly thinner than shafts of falcigers of same fascicle, with short subdistal spines, distally bidentate, teeth of similar size, progressively more triangular posteriorwards (Figs 4F, 4G and 5C); ventral simple chaetae only present in posteriormost parapodia, sigmoid, as thick as shafts of falcigers of same fascicle, with short subdistal spines, distally bidentate, distal tooth larger (Figs 4H and 5D). Anterior parapodia with two aciculae each, one of which straight, with acute tip, another subdistally inflated and twisted, with long, thin, needle-like tip (Fig 5E); from midbody onwards, single acicula per parapodium, of latter type, stouter posteriorly (Fig 5F). Semicircular pygidium with paired anal cirri, similar in shape to posterior body dorsal cirri, but longer. Pharynx through 3–4 segments; tooth relatively long, rhomboidal to ovate, slightly away from anterior margin of pharynx; proventricle extending for 2–3 chaetigers, with 19–22 rows of muscle cells.

#### Reproduction

Some specimens were found brooding eggs dorsally, from around chaetigers 8–20, with 1 or 2 eggs per chaetiger.

#### Remarks

Members of *Salvatoria nitiduloides* **n. sp.** are characterized by the length pattern of anterior body dorsal cirri. These animals are similar to *S*. *clavata* from the Iberian Peninsula, according to the redescription provided by [7], in overall body shape and by the falciger blades having distal tooth larger than the subdistal one throughout. San Martín [7] noticed different morphotypes of *S*. *clavata* in his material and considered those more than just intraspecific variation, suggesting the occurrence of a species complex. *Salvatoria clavata* is characterized by having parapodial lobes papillated, with at least three small papillae; blades of falcigers on anterior body with dorso-ventral gradation in length, 19–12 μm long, dorso-ventral gradation in length absent on posterior body, with blades 11–12 μm long; blades of falcigers with short, straight spines on anterior body and with stouter, short, straight spines on posterior body; dorsal simple chaetae bidentate, with short subdistal tooth; and single acicula per parapodium, acuminate [7]. Specimens of *S*. *nitiduloides* **n. sp.** described herein differ from the description of *S*. *clavata* from the Iberian Peninsula by having smooth parapodial lobes, without papillae; longer blades of falcigers, with dorso-ventral gradation in length throughout, 22–8 μm, 25–12 μm and 24–11 μm long on anterior, mid- and posterior body parapodia, respectively; longer spinulation on the cutting edges of the blades of dorsalmost falcigers throughout; dorsal simple chaetae with both teeth triangular, similar in length; and anterior parapodia with two aciculae with acute tip each, one of which straight, another subdistally inflated and twisted.

*Salvatoria nitiduloides* **n. sp.** is similar to *S*. *nitidula* [18, 19] and *S*. cf. *nitidula* characterized above, in overall shape of body, antennae and cirri throughout. However, members of *S*. *nitiduloides* **n. sp.** have the median antenna inserted slightly posteriorly, between posterior pair of eyes; dorsal peristomial cirri similar in size to median antenna; dorsal cirri from chaetigers 4 and 5 as long as those of the chaetiger 1, and subsequent dorsal cirri shorter; falciger blades with distal tooth larger than the subdistal one throughout; falciger blades shorter, 22–8 μm, 25–12 μm and 24–11 μm long on anterior, mid- and posterior body parapodia, respectively. In contraposition, both *S*. *nitidula* and *S*. cf. *nitidula* have median antenna inserted between the anterior pair of eyes; dorsal peristomial cirri shorter median antenna; dorsal cirri from chaetiger 1 longer than remaining; falciger blades with distal tooth larger than subdistal one on anterior and midbody parapodia, teeth about same size on posterior body; and falciger blades longer, 30–10 μm on anterior and midbody parapodia, respectively. Also, *S*. *nitiduloides* **n. sp.** also differs from *S*. cf. *nitidula* by having ventral simple chaetae subdistally spinulated under SEM and proventricle shorter, extending for up to 3 segments.

#### Type locality

Ilha de Itamaracá, Pernambuco, Brazil (SW Atlantic).

#### Distribution

South Atlantic, Brazil: states of Paraíba and Pernambuco (Fig 1A and 1B).

***Salvatoria marielleae* Nascimento, Fukuda & Paiva n. sp.**

urn:lsid:zoobank.org:act:4A89DBDD-4BD0-4743-B965-6EB45A017BCB

**Fig 7:**
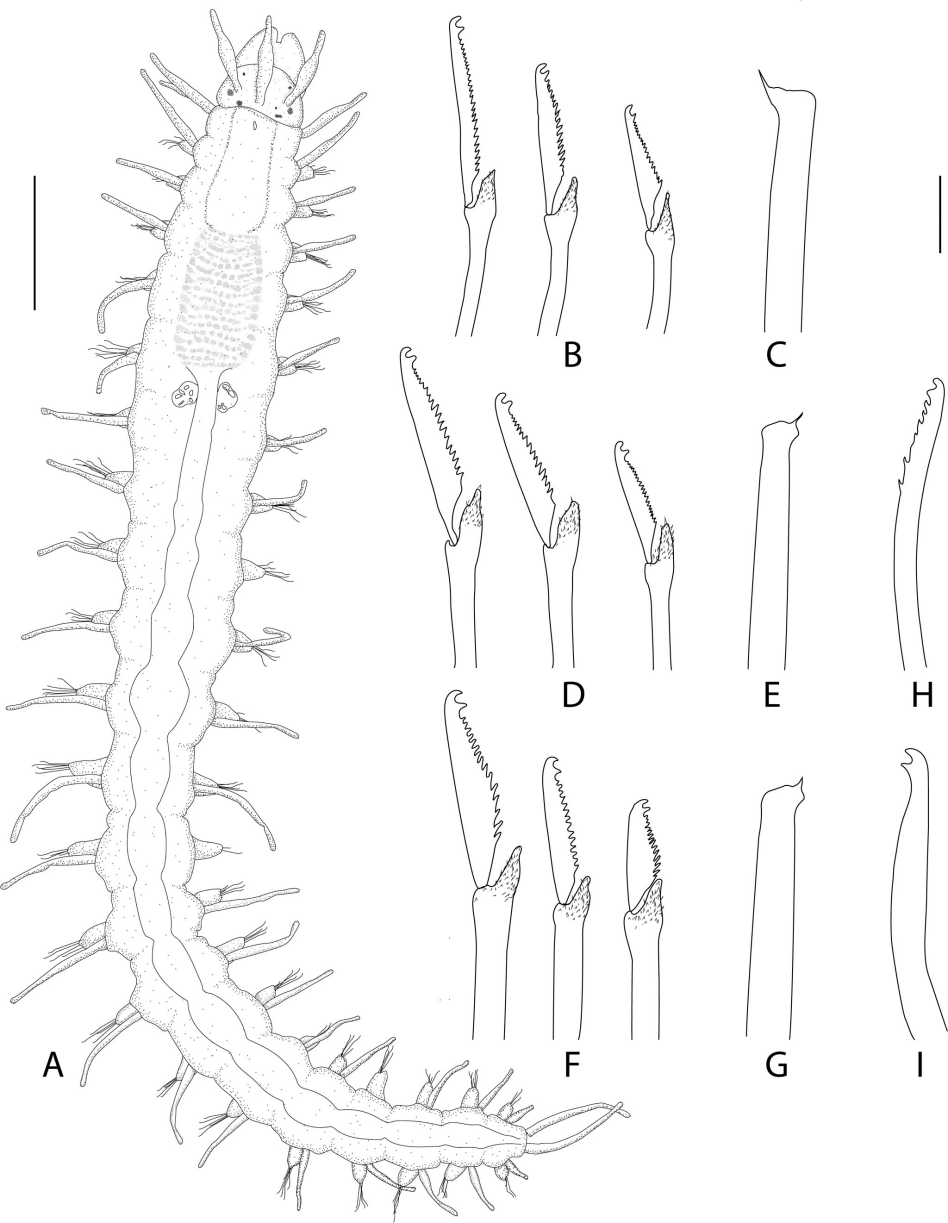
*Salvatoria marielleae* n. sp. (A). Entire body, dorsal view; (B), (D), (F). Falcigers, anterior, mid- and posterior body, respectively; (C), (E), (G). Aciculae, anterior, mid- and posterior body, respectively; (H). Dorsal simple chaeta; (I). ventral simple chaeta. Scale bar: A, 0.18 mm; B–I, 6 μm.

**Fig 8:**
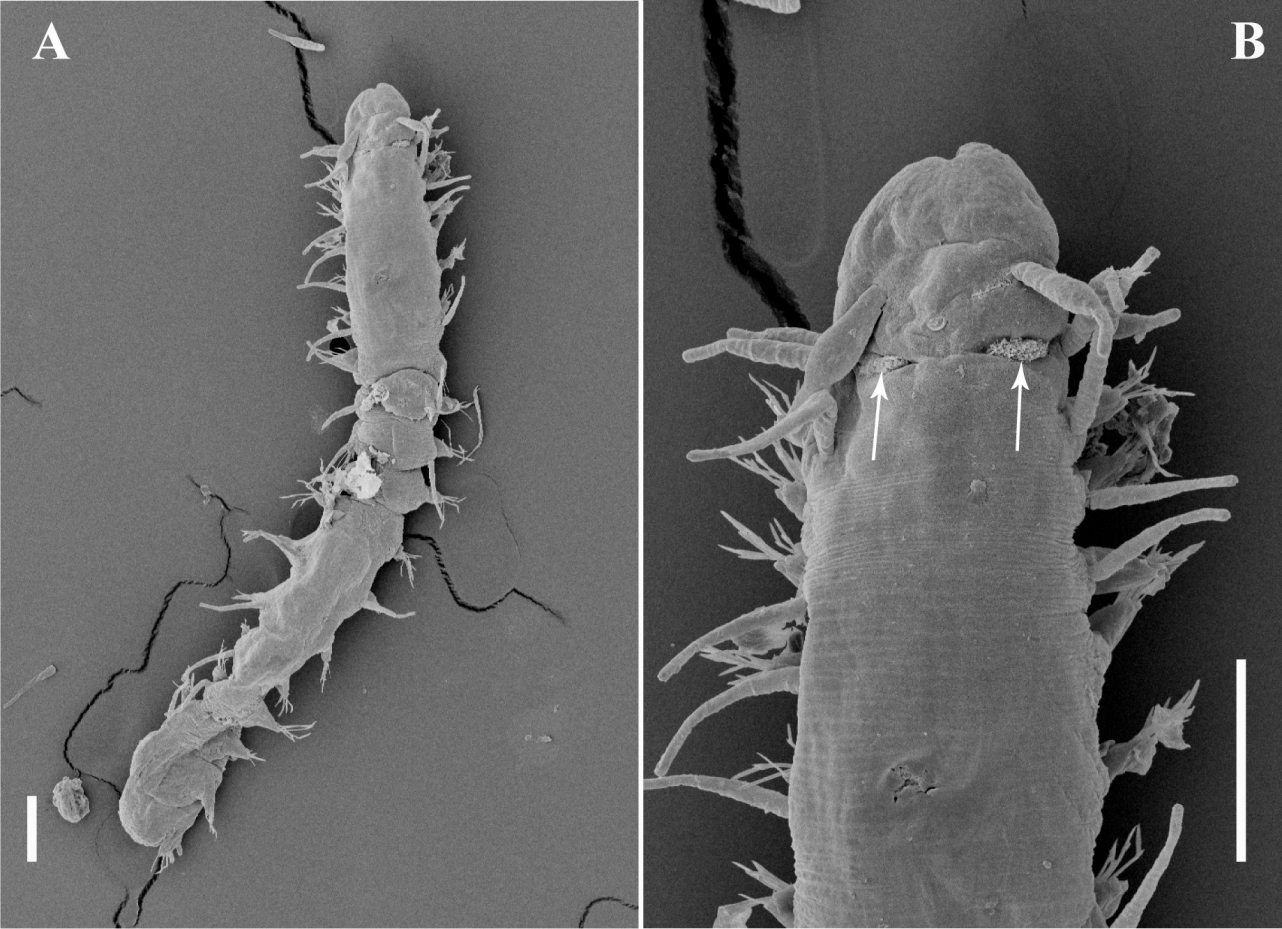
*Salvatoria marielleae* n. sp., paratype 3 (MZUSP 3593), SEM. (A). Incomplete specimen, dorsal view; (B). Anterior body, dorsal view. Arrows pointing to ciliated nuchal organs. Scale bars: A–B, 100 μm.

**Fig 9:**
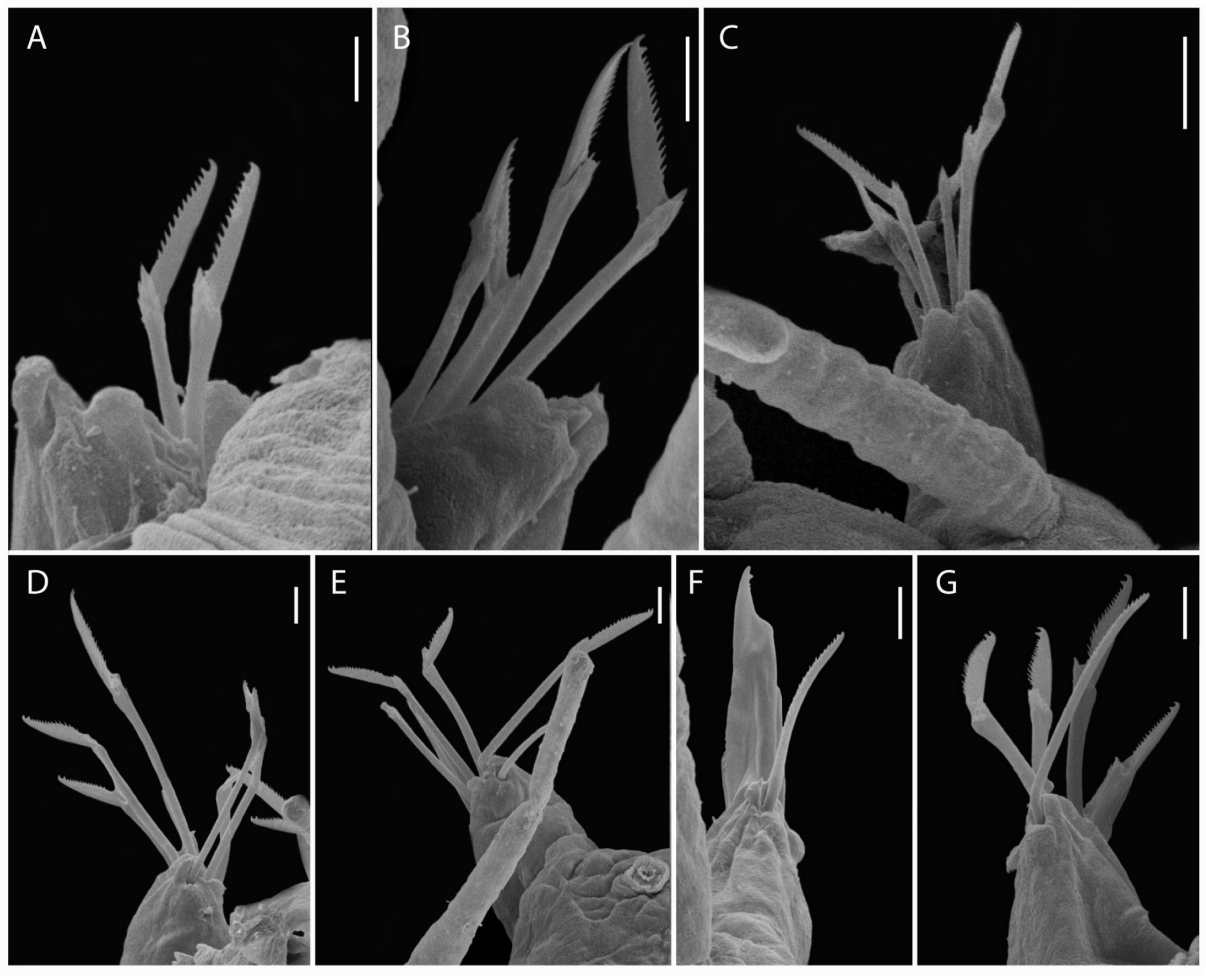
*Salvatoria marielleae* n. sp., paratype 3 (MZUSP 3593), SEM. (A–C). Falcigers, anterior body; (D–E). Falcigers, midbody; (F–G). Falcigers and dorsal simple chaetae, posterior body. Scale bars: A–B, 4 μm; C, 10 μm; D–G, 6 μm.

Figs 6A, 7–9, Tables 1 and 2.

**Table 2:** Morphological variations of selected characters among specimens in the type series of *Salvatoria marielleae* n. sp.

Selected characters	Holotype MNRJP1918	Paratype 1 MZUSP3592	Paratype 2 MNRJP1919	Paratype 3 MZUSP3593
**Body length/width (mm)**	1.9 / 0.18	2.3 / 0.18	1.7 / 0.17	1.8 / 0.18
**Number of chaetigers**	21	22	21	20
**Number of proventricle segments/muscle cell rows**	2 / 18	2 / 18	2.5 / 18	2.5 / 19
**Number of pharynx segments**	3	3	3	3
**Length of falciger blades (μm)**				
**Anterior body**	21‒10	20‒10	20‒9	21‒10
**Midbody**	21‒12	20‒10	20‒10	21‒14
**Posterior body**	20‒10	18‒10	20‒10	20‒10
**Dorsal/ventral simple chaetae starting from (chaetiger)**	5 / 17	6 / 20	5 / 19	5 / —

#### Type series

*Holotype*: Project ‘*Island Syllids*’—Fernando de Noronha Island, Praia do Atalaia (03°50’55”S 32°24’45”W), 0.5 m deep, on red algae: (MNRJP 1918), coll. 23 April 2016. Paratypes: Fernando de Noronha Island, Praia do Atalaia (03°50’55”S 32°24’45”W), 0.5 m deep, on red algae–Paratype 1 (MZUSP 3592), Paratype 2 (MNRJP 1919), Paratype 3 (MZUSP 3593, mounted for SEM), coll. 23 April 2016. Morphometric data of specimens from the type series are given in Table 2.

#### Other material examined

Project ‘*ProTrindade*’—Trindade and Martin Vaz Archipelago, Ilha de Martin Vaz (20°30’45”S 29°18’21”W), 12m depth: 1 specimen (MZUSP 2325), coll. 24. Jul 2013.

#### Comparative material examined

*Brania brevypharyngea* Banse, 1972 [33]. North Pacific Ocean—USA, Washington, San Juan Island, False Bay (48°28’56”N 123°03’52”W), ~1.5 m deep, among aggregate of *Bansella oregonica* (Banse, 1956) [34] tubes: Holotype (USNM 40711), leg. & det. K. Banse, 29 June 1969.

#### Description

**Fig 10:**
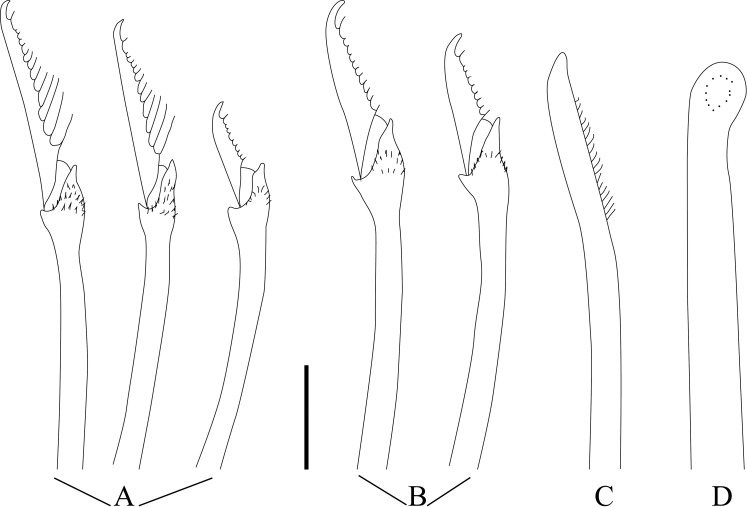
*Salvatoria neapolitana*. (A). Falcigers, anterior body and midbody; (B). Falcigers, posterior body; (C). Dorsal simple chaeta; (D). Acicula. Scale bar: 10 μm.

Small-sized species, longest specimen examined Paratype 1 (MZUSP 3592), 2.3 mm long, 0.18 mm wide, with 22 chaetigers; holotype complete (MNRJP 1918) (Figs 6A and 7A), 1.9 mm long, 0.18 wide, with 21 chaetigers (Table 2). Palps with approximately same length as prostomium, reniform to triangular, dorsally united by membrane for ¾ of their length (Figs 7A, 8A and 8B). Prostomium subpentagonal (Figs 7A, 8A and 8B), with two pairs of eyes in open trapezoidal arrangement, anterior pair slightly larger than posterior one, and pair of anterior eyespots (Fig 7A). Antennae spindle-shaped, all of same length; median antenna inserted slightly anterior to posterior pair of eyes, sometimes exceeding length of palps and prostomium, lateral antennae inserted in front of anterior pair of eyes, away from anterior margin of prostomium (Figs 7A and 8B). Two ciliated nuchal organs dorsolaterally between prostomium and peristomium (Fig 8A and 8B). Peristomium with two pairs of peristomial cirri; dorsal peristomial cirri with approximately same length as antennae, ventral peristomial cirri ⅓ shorter than dorsal cirri (Figs 7A and 8B). Peristomial and dorsal cirri throughout digitiform, distally tapered, with bases slightly thickened; dorsal cirri of chaetiger 1 longer than those of chaetigers 2‒8; midbody dorsal cirri all similar in length or slightly shorter than those of chaetiger 1 (Figs 7A and 8A). Ventral cirri digitiform, shorter than parapodial lobes. Parapodia with 7‒5 falcigers each throughout; falciger blades spinulated, with relatively short, coarse spines, and strongly bidentate, with teeth similar in size and rounded space inbetween (Figs 7B, 7D, 7F, 9A–9E and 9G); blades on mid- and posterior body chaetigers with proximal part slightly broader, tapering distally (Figs 7F, 9D, 9E and 9G); blades of falcigers with dorsoventral gradation in length (Table 2), 21‒9 μm long on anterior body (Figs 7B and 9A‒9C), 21‒10 μm long on midbody (Figs 7D, 9D and 9E), and 20‒10 μm long on posterior body chaetigers (Figs 7F and 9G). Dorsal simple chaetae present from proventricle level (Table 2), thinner than falciger shafts, slightly sigmoid, bidentate (Fig 7H), with subdistal tooth about same size or slightly larger than distal one, and spinulated, serrate in appearance (Fig 9F and 9G). Ventral simple chaetae present only on posterior body parapodia (Table 2), about as thick as falcigers shafts, strongly sigmoid and bidentate, with teeth about same size, apparently smooth (Fig 7I). Single acicula per parapodium throughout, subdistally inflated and curved, with acute tip pointing upwards (Figs 7C, 7E and 7G). Pygidium with pair of anal cirri, about same length or longer than dorsal cirri of posterior chaetigers (Figs 6A and 7A). Pharynx through 3 segments, anterior margin smooth; pharyngeal tooth ovate, located near anterior margin of pharynx; proventricle through ~2.5 segments, with 18‒19 muscle cell rows (Figs 6A and 7A and Table 2).

#### Remarks

*Salvatoria marielleae* **n. sp.** has the prostomium slightly broader than palps; antennae spindle-shaped and peristomial and dorsal cirri tapered with slightly stouter bases; dorsal peristomial cirri longer than ventral ones; dorsal cirri of chaetiger 1 longer than those of chaetigers 2‒8; midbody dorsal cirri about same length or slightly shorter than those of chaetiger 1.

*Salvatoria nitidula* [18] is the species most similar to *S*. *marielleae* **n. sp.** by the overall body shape and morphology of antennae and chaetae. However, *S*. *nitidula* has palps slightly broader than prostomium; antennae and peristomial cirri about same shape; both dorsal and ventral peristomial cirri similar in length; dorsal cirri about same length as peristomial cirri or the width of corresponding segment [18]. Specimens of *S*. *nitidula* from Cuba, Bermuda, and Gulf of Mexico [19], have falciger blades bidentate with distal tooth slightly larger than subdistal one in anterior and midbody parapodia, whereas *S*. *marielleae* **n. sp.** has teeth of similar sizes throughout. The specimens described by [19] have stronger dorsoventral gradation in the length of the blades, 30‒14 μm on anterior body and 25‒15 μm on posterior body, whereas *S*. *marielleae* **n. sp.** presents falciger blades with 21‒9 μm on anterior body, 21‒10 μm on midbody, and 20‒10 μm on posterior body. In addition, specimens of *S*. *marielleae* **n. sp.** have dorsal simple chaetae from the proventricle level and ventral simple chaetae smooth under optical microscope, whereas the specimens of *S*. *nitidula* analysed by [19] have dorsal simple chaetae from the first chaetigers onwards, and ventral simple chaetae slightly spinulated. Finally, *S*. *nitidula* presents two aciculae per parapodium on anterior body chaetigers, one of which straight, distally pointed, slightly protruding from parapodial lobes, the other subdistally inflated, twisted, with acute tip, while *S*. *marielleae* **n. sp.** presents only one acicula per parapodium throughout, of the latter type.

The specimens of *S*. cf. *nitidula* characterized herein differ from *S*. *marielleae* **n. sp.** in having median antenna longer than lateral ones; lateral antennae inserted closer to anterior margin of the prostomium; antennae, peristomial and dorsal cirri spindle-shaped to subulated; dorsal cirri of chaetiger 1 longer the remaining; and blades of falcigers longer, with up to 30 μm and 25 μm on anterior and posterior body, respectively. In contraposition, *S*. *marielleae* **n. sp.** has all antennae of similar size; lateral antennae inserted away from the anterior margin of prostomium; peristomial and dorsal cirri slightly different from the antennae, digitiform, distally tapered, with bases slightly stouter; falciger blades shorter, with up to 21 μm and 20 μm long on anterior and posterior parapodia, respectively, with teeth of similar size throughout and rounded space inbetween; ventral simple chaetae smooth; single acicula per parapodium throughout, subdistally inflated, with acute tip.

*Salvatoria nitiduloides* **n. sp.** is also similar to *S*. *marielleae* **n. sp.**, by the overall body shape and by having falciger blades and dorsal and ventral simple chaetae bidentate and spinulated, and aciculae with similar morphology. However, *S*. *nitiduloides* **n. sp.** differs from *S*. *marielleae* **n. sp.** by having antennae and dorsal cirri throughout spindle-shaped to subulated; falciger blades with distal tooth larger than subdistal one and oblique space inbetween; ventral simple chaetae subdistally spinulated; and two aciculae per parapodium on anterior body chaetigers, one thin, straight, the other subdistally inflated, with acute tip. Differently, *S*. *marielleae* **n. sp.** has antennae typically spindle-shaped; dorsal cirri morphologically different from antennae, digitiform, distally tapered, with bases only slightly stouter; falciger blades with teeth of similar size and rounded space inbetween; ventral simple chaetae smooth; and single acicula per parapodium throughout, subdistally inflated and twisted, with acute tip.

*Salvatoria brevipharyngea* (Banse, 1972) [33] and *S*. *euritmica* (Sardá, 1984) [27] are also similar to *S*. *marielleae* **n. sp.** in the overall body shape and morphology of antennae, cirri and chaetae, especially regarding the bidentate falciger blades, with teeth of similar sizes and rounded space between teeth. However, *S*. *brevipharyngea*, described from the North Pacific coast of the United States, and so far only known from that area [33, 35], is a larger species, up to 3 mm in length and with 31 chaetigers (against up to 2.3 mm long, with 22 chaetigers, as in members of *S*. *marielleae* **n. sp.**); it also has a slightly shorter pharynx (~2.5 segments) and slightly larger proventricle (~3.5–4.5 segments, with 20–25 muscle cell rows) than *S*. *marielleae* **n. sp.**, which shows the pharynx extending for three segments and the proventricle, for 2–2.5, with 18–19 muscle cell rows. Finally, the aciculae in *S*. *brevipharyngea* are subdistally inflated and slightly oblique, with rounded tip, different from the aciculae with filiform, needle-like tips, as common in the genus and present in *S*. *marielleae* **n. sp.**

*Salvatoria euritmica*, described and with occurrences throughout the Mediterranean and also occurring in the North Atlantic European coast, Cuba, Brazil and Australia [7, 17], is also a larger species than *S*. *marielleae* **n. sp.**, up to 4 mm in length and with 29 chaetigers. It also differs from *S*. *marielleae* **n. sp.** in having all dorsal cirri of similar length throughout, except for those from chaetiger 1, which are longer; falcigers with longer blades (31–9 μm long, against 21–9 μm long in *S*. *marielleae* **n. sp.**); and in presenting up to two aciculae per anterior body parapodium, one of which subdistally inflated and twisted, similar to the single acicula present in each parapodium of *S*. *marielleae* **n. sp.**, but without the acute, needle-like tip, the other acicula being nearly straight, subdistally inflated, with tapering tip. It is worth mentioning that, as specimens of *S*. *euritmica* from different localities show slight differences among them [7, 17], the comparison above was made based on European specimens. Thus, this would be another good target for focused taxonomic investigations, to check whether it is a case of species complex.

#### Etymology

This species is named after Marielle Franco da Silva, known as Marielle Franco (27.Jun.1979–14.Mar.2018) a Brazilian black woman, sociologist, politician, feminist and human rights advocate, murdered after dedicating her life to assist the minorities in Brazil. Marielle, Presente!

#### Type locality

Fernando de Noronha island, Pernambuco, Brazil (SW Atlantic).

#### Distribution

South Atlantic, Brazil: Fernando de Noronha and Trindade islands (Figs 1A, 1D and 1E).

***Salvatoria neapolitana* (Goodrich, 1930) [23]**

Fig 10 and Table 1.

*Pionosyllis neapolitana* Goodrich, 1930 [23]: 651–666, Figs 1–12.

*Pionosyllis subterranea* Hartmann-Schröder, 1956 [25]: 89–91, Figs 6–9.

*rania subterranea*. Westheide, 1974a [24]: 10–14, Abb. 6; 1974b [36]: 279–281, Abb. 40.

*Grubeosyllis neapolitana*. Jiménez *et al*., 1994 [37]: 52–53, Figs 1–2.

*Salvatoria neapolitana*. San Martín, 2003 [7]: 182–184, Fig 94.

#### Examined material

Project ‘*BIOTA*’—State of São Paulo, Ubatuba, Praia de Picinguaba, (23°23’49’’S 45°58’00”W), 12.1 m deep: 1 specimen, coll. 21 August 2001; (23°24’154’’S 44°59’540’’W), 7.9 m deep: 2 specimens (MZUSP 4276), coll. 21 August 2001; (23°27’033’’S 45°03’013’’W), 5 m deep: 3 specimens, coll. 21 August 2001; (unknow date): 1 specimen, coll. 12 November 2001; (unknow date): 4 specimens, coll. 12 Nov. 2001; on *Sargassum* sp. (unknow date): 4 specimens; interstitial in sand: 20 specimens (MZUSP 4275), coll. 22 August 2001. State of São Paulo, São Sebastião, Praia de São Francisco (23°44’54’’S 45°24’33’’W), interstitial in sand: 36 specimens (MZUSP 4271–4273), coll. 27 May 2001; 59 specimens (MZUSP 4277–4280), coll. 15 October 2001; 1 specimen (MZUSP 4274), coll. 14 January 2002.

#### Description

Small-sized body, longest specimen analysed with 23 chaetigers, 1.8 mm long, 0.15 mm wide (Table 1). Short palps, reniform to triangular. Prostomium subpentagonal, with 2 pairs of eyes in trapezoidal arrangement; median antenna inserted between posterior pair of eyes, much longer than palps and prostomium together, lateral antennae inserted in front of anterior pair of eyes, 2/3 length of median antenna. Peristomium as long as or slightly shorter than subsequent chaetigers; dorsal peristomial cirri of about same length as median antenna, ventral peristomial cirri almost same length as lateral antennae. Antennae, peristomial and dorsal cirri throughout with similar morphology, with bases slightly inflated, tapering distally, smooth to strongly wrinkled; dorsal cirri present on all chaetigers, those of chaetiger 1 longer than remaining, progressively shorter towards posterior body; ventral cirri digitiform, about same length as parapodial lobes. Anterior and midbody parapodia with ca. 10 falcigers each, posterior body parapodia with 2–5 falcigers each; falciger shafts subdistally spinulated, spines more conspicuous on dorsalmost chaetae of anterior body and midbody chaetigers (Fig 10A and 10B); blades sub-bidentate, with thin, spine-like subdistal tooth, and strongly spinulated, with oblique spines on dorsalmost blades of anterior parapodia, progressively smaller and straighter ventralwards (Fig 10A and 10B); blades with conspicuous dorsoventral gradation in length, 19–9 μm long throughout. Dorsal simple chaetae present on all chaetigers, sigmoid, unidentate, subdistally spinulated (Fig 10C); ventral simple chaetae present on posteriormost chaetigers, same morphology as dorsal simple chaetae, about half thickness of falcigers shafts. Single acicula per parapodium throughout, distally hollow, with tip slightly inflated and oblique (Fig 10D). Anal cirri longer than posterior body dorsal cirri. Pharynx extending through 3 chaetigers, anterior margin surrounded by soft papillae and conical tooth at ^1^/₃ of its length; proventricle through ~ 3.5 segments, with ~ 20 muscle cell rows.

#### Remarks

*Salvatoria neapolitana* has rounded, distally hollow aciculae, unusual for *Salvatoria* but characteristic of the genus *Brania* Quatrefages, 1866 [15]. Nevertheless, this species is undoubtfully placed in *Salvatoria* for a characteristic set of characters, such as the morphology of the dorsal simple chaetae, position of pharyngeal tooth and length of pharynx and proventricle, the latter without a conspicuous midline formed by the arrangement of the muscle cell rows, typical of *Brania*.

*Salvatoria neapolitana* resembles *S*. *vieitezi* in the morphology of falciger blades–bidentate, with subdistal tooth clearly smaller than distal one, conspicuously spinulated, with some spines directed upwards, and with dorsoventral gradation in length. Conversely, *S*. *vieitezi* differs from *S*. *neapolitana* by having clearly bidentate blades, although with subdistal tooth slightly smaller than distal one, and marked differences between blades of dorsalmost and ventralmost falcigers within same fascicle, the former with long spines directed upwards, the latter with short, straight spines; additionally, *S*. *vieitezi* presents dorsal, transverse dark-red stripes on each segment and has acuminated aciculae, with acute and filiform tips, as typical of the genus [7].

Another species with falciger blades with small, spine-like subdistal tooth is *S*. *kerguelensis* McIntosh, 1885 [14], which also shares with *S*. *neapolitana* the size of the antennae, peristomial and dorsal cirri throughout, and proventricle proportions. However, *S*. *kerguelenesis* differs from *S*. *neapolitana* in having dorsal simple chaetae slightly less sigmoid than in animals belonging to the latter species, and aciculae subdistally inflated and slightly twisted, with acute tip, as typical of *Salvatoria* [17] and different from the distally hollow aciculae of *S*. *neapolitana*.

*Salvatoria heterocirra* also has falcigers with bidentate and spinulated blades and aciculae with distally inflated, hollow tip. Conversely, *S*. *heterocirra* differs from *S*. *neapolitana* in having dorsal simple chaetae conspicuously more sigmoid than in *S*. *neapolitana* and falciger blades with teeth of similar size, with characteristic rounded space inbetween [28].

The specimens examined herein differ from the Mediterranean ones (lectotype and paralectotype assigned by [37]) by having shorter falciger blades, 32–12 μm and 26–12 μm long on anterior and posterior parapodia, respectively, for Mediterranean specimens [7, 37], and blades 19–9 μm long throughout in the specimens herein examined.

#### Type locality

Naples Bay, Italy, Mediterranean Sea, Atlantic Ocean.

#### Distribution

Pacific Ocean: Galapagos islands; Atlantic Ocean: Mediterranean Sea, France, Spain, Italy, Canary Islands, Brazil (State of São Paulo) (Fig 1A–1C).

***Salvatoria ypsiloides* Nascimento, Fukuda & Paiva n. sp.**

urn:lsid:zoobank.org:act:BD9C46A5-4384-45B6-9413-C4FED88513B8

**Fig 11:**
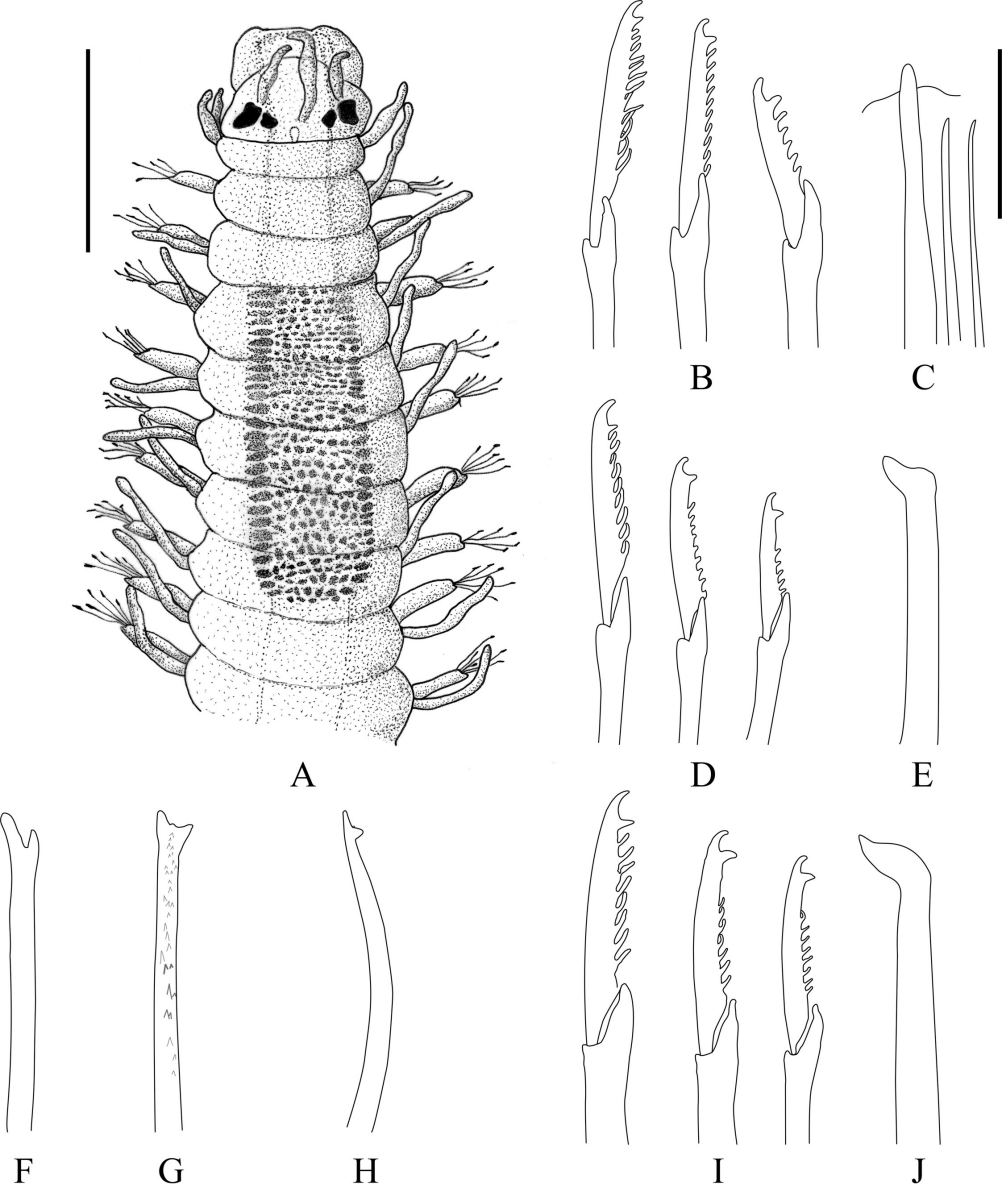
*Salvatoria ypsiloides* n. sp. (A). Anterior body, dorsal view; (B), (D), (I). Falcigers, anterior, mid- and posterior body, respectively; (C), (E), (J). Aciculae, anterior, mid- and posterior body, respectively; (F–G). Dorsal simple chaetae, mid- and posterior body, respectively; (H). Ventral simple chaeta. Scale bars: A, 0.14 mm; B–J, 10 μm.

**Fig 12:**
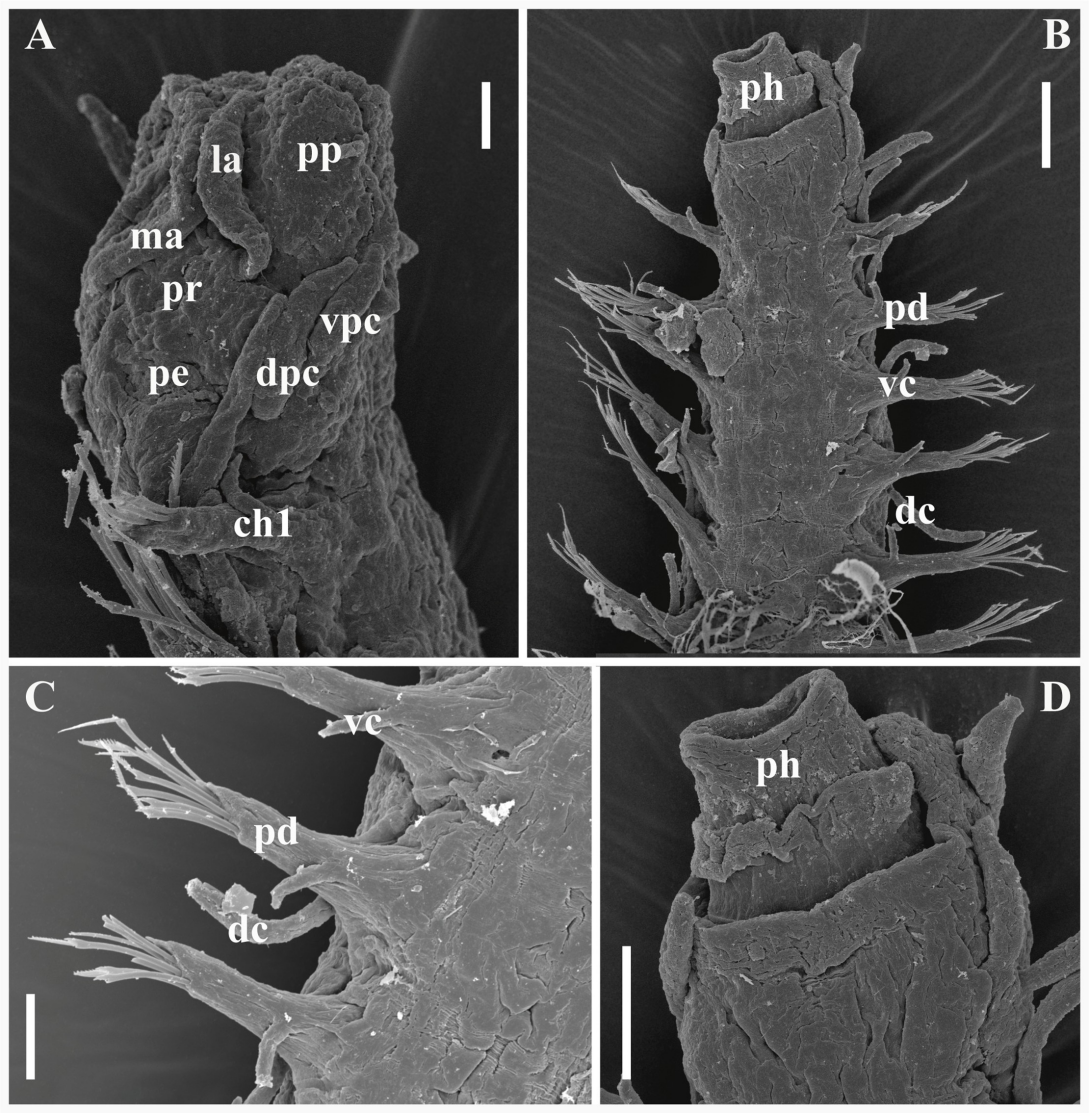
*Salvatoria ypsiloides* n. sp., SEM. (A). Anterior body, dorso-lateral view; (B). Anterior body, ventral view; (C). Anterior parapodia, ventral view; (D). Prostomium and peristomium (pharynx everted), ventral view. **ch1** –chaetiger 1; **dc**–dorsal cirrus; **dpc**–dorsal peristomial cirrus; **la**–lateral antenna; **ma**–median antenna; **pd**–parapodium; **pe**–peristomium; **ph**–pharynx (everted); **pp**–palps; **pr**–prostomium; **vc**–ventral cirrus; **vpc**–ventral peristomial cirrus. Scale bars: A, D, 10 μm; B, 50 μm; C, 20 μm.

**Fig 13:**
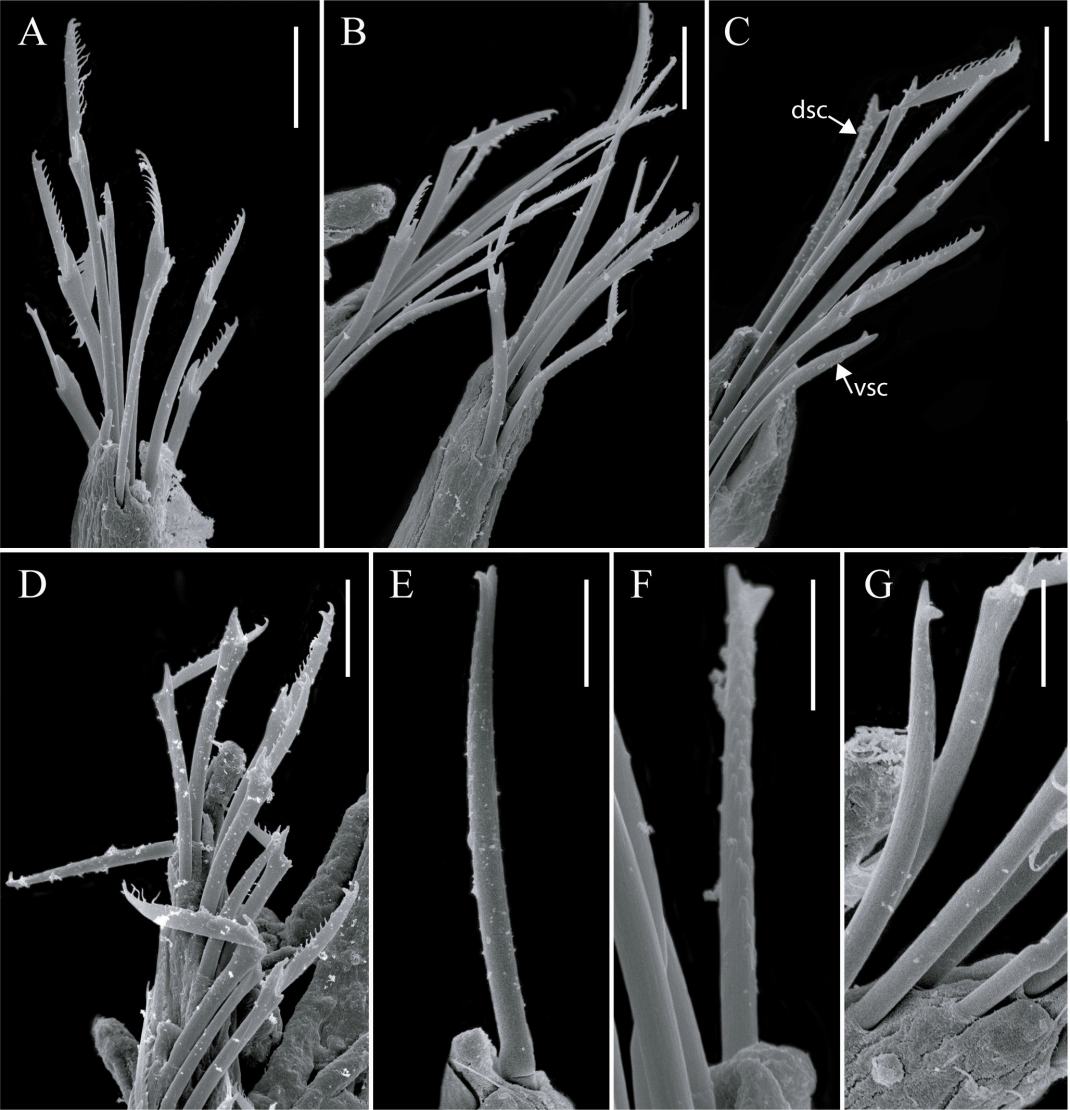
*Salvatoria ypsiloides* n. sp., SEM. (A–B). Falcigers, anterior and midbody, respectively; (C). Falcigers, dorsal and ventral simple chaetae, midbody; (D). Falcigers, posterior body; (E–F). Dorsal simple chaetae; (G). Ventral simple chaeta, posterior body. Scale bars: A–E, G, 10 μm; F, 2 μm. **dsc**–dorsal simple chaeta; **vsc**–ventral simple chaeta.

Figs 11–13, Tables 1 and 3.

**Table 3:** Morphological variation of selected characters among specimens in the type series of *Salvatoria ypsiloides* n. sp.

Selected characters	Holotype MNRJP1915	Paratype 1 MZUSP3590	Paratype 2 MNRJP1916	Paratype 3 MZUSP3591	Paratype 4 MNRJP1917
**Body length/width (mm)**	1.65 / 0.14	2.2 / 0.12	1.37 / 0.12	1.42 / 0.13	1.35 / 0.10
**Number of chaetigers**	30	32	26 (incomplete)	28	25
**Number of proventricle segments/muscle cell rows**	5 / 29	5 / 27	5.5 / 26	5.5 / 26	4.5 / 26
**Number of pharynx segments**	5	4.5	4	4	4.5
**Length of falciger blades (μm)**					
** Anterior body**	14‒8	15‒7.4	18‒9	18‒8	17‒9
** Midbody**	16‒9	18‒10	18‒9	18‒9	16‒8
** Posterior body**	17‒10	18‒10	16‒8	18‒8	19‒8
**Dorsal/ventral simple chaetae starting from (chaetiger)**	1 / 16	1 / 15	1 / 16	1 / 11	1 / 17

#### Type series

*Holotype*: Project ‘*Island Syllids*’—Fernando de Noronha Island, Praia do Atalaia (03°50’55’’S 32°24’45’’W), 0.5 m deep, on algae: (MNRJP 1915), coll. 23 April 2016. Paratypes: Fernando de Noronha Island, Praia do Atalaia (03°50’55’’S 32°24’ 45’’W), 0.5 m deep, on algae: Paratype 1 (MZUSP 3590), Paratype 2 (MNRJP 1916), Paratype 3 (MZUSP 3591), Paratype 4 (MNRJP 1917) and Paratype 5, mounted for SEM (MNRJP 1984), all coll. 23 April 2016. Project ‘*Habitats*’—State of Rio de Janeiro, Campos Basin, soft bottom: 21°44’19”S 40°17’15”W, 50 m deep: 5 paratypes (MZUSP 3779), coll. 08 July 2009. Morphometric data of selected specimens from the type series are given in Table 2.

#### Additional material examined

Project ‘*Island Syllids*’—Fernando de Noronha Island, Praia do Atalaia (03°50’55”S 32°24’45”W), 0.5 m deep, on algae: 6 specimens (MNRJP 1917), coll. 23 April 2016. Project ‘*AMBES*’—State of Espírito Santo, Espírito Santo Basin, soft bottom: 18°36’31”S 39°9’33”W, 39 m deep: 1 specimen (MZUSP 3731), coll. 17 January 2012; 19°18’6”S 39°23’23”W, 38 m deep: 2 specimens (MZUSP 3741), coll. 15 July 2013; 19°47’32”S 39°43’15”W, 41 m deep: 4 specimens (MZUSP 3746), coll. 15 December 2010; 19°53’27”S 39°32’59”W, 970 m deep: 1 specimen (MZUSP 3733), coll. 28 June 2013; 20°10’2”S 40°8’31”W, 25 m deep: 2 specimens (MZUSP 3729), coll. 20 January 2012; 20°12’20”S 39°57’59”W, 50 m deep: 14 specimens (MZUSP 3730), coll. 20 January 2012; 20°34’47”S 40°11’31”W, 41 m deep: 1 specimen (MZUSP 3725), coll. 12 July 2013; 20°34’53”S 40°6’27”W, 50 m deep: 1 specimen (MZUSP 3728), coll. 21 January 2012; 21°2’45”S 40°32’29”W, 25 m deep: 1 specimen (MZUSP 3723), coll. 22 January 2012; 21°4’1”S 40°18’50”W, 49 m deep: 3 specimens (MZUSP 3724), coll. 22 January 2012. Project ‘*Habitats*’—State of Rio de Janeiro, Campos Basin, soft bottom: 21°11’0”S 40°28’27”W, 26 m deep: 15 specimens (MZUSP 3771), coll. 05 March 2009; 21°44’18”S 40°17’14”W, 49 m deep: 35 specimens (MZUSP 3769), coll. 09 March 2009; 21°59’4”S 40°25’12”W, 53 m deep: 1 specimen (MZUSP 3777), coll. 06 July 2009; 22°6’56”S 40°38’58”W, 53 m deep: 5 specimens (MZUSP 3758), coll. 26 February 2009; 22°7’43”S 40°18’46”W, 73 m deep: 1 specimen (MZUSP 3763), coll. 24 February 2009; 22°12’53”S 40°51’12”W, 52 m deep: 2 specimens (MZUSP 3756), coll. 26 February 2009; 22°46’54”S 41°3’33”W, 78 m deep: 1 specimen (MZUSP 3772), coll. 02 July 2009; 22°55’7”S 42°0’49”W, 29 m deep: 1 specimen (MZUSP 3753), coll. 28 February 2009.

#### Description

Small-sized species, longest specimen examined Paratype 1 (MZUSP 3590), ca. 2.2 mm long, 0.12 mm wide, with up to 32 chaetigers; holotype complete (MNRJP 1915), 1.65 mm long, 0.14 mm wide, with 30 chaetigers (Figs 6B, 11A and Table 3). Palps shorter than prostomium (Fig 11A), united by dorsal membrane, with small distal notch (Figs 6B and 11A). Prostomium subpentagonal, with two pairs of eyes in wide open trapezoidal arrangement, nearly aligned, anterior pair larger than posterior one; eyespots small, frequently inconspicuous, close to anterior margin of prostomium (Fig 11A); antennae spindle shaped to digitiform, slightly enlarged at mid-length; median antenna inserted between posterior pair of eyes, sometimes slightly exceeding length of palps; lateral antennae shorter, inserted in front of anterior pair of eyes, at anterior margin of prostomium (Figs 11A and 12A). Peristomium slightly shorter than anterior chaetigers; dorsal peristomial cirri about as long as lateral antennae (Figs 11A and 12A); ventral peristomial cirri with ¾ length of dorsal peristomial cirri. Dorsal cirri all about same length throughout, elongate, digitiform, almost of uniform width along their length (Figs 11A and 12B), frequently with hollow appearance more pronounced close to tip, sometimes distally slightly swollen. Ventral cirri digitiform inserted on basal ⅓ of parapodial lobes, not reaching their tips (Fig 12C); some ventral cirri distally hollow, similar to dorsal cirri. Parapodia with 5–7 compound falcigers each throughout; blades strongly bidentate, on anterior body distal tooth slightly shorter than subdistal one (Figs 11B and 13A), teeth about same size on mid- and posterior body chaetigers (Figs 11D, 11I and 13D); angle between teeth wider and subdistal tooth progressively slightly larger than distal one ventralwards within each fascicle (Fig 11B, 11D and 11I); blades spinulated, dorsalmost falcigers with larger and thickened spines, usually directed upwards, reaching base of subdistal tooth in a few dorsalmost falcigers, spines short, straight, in remaining falcigers (Figs 11B, 11D, 11I, 13A and 13C and 13D); blades with marked dorsoventral but inconspicuous antero-posterior gradation in length (Table 3), measuring 18‒7.4 μm long on anterior body (Figs 11B and 13A), 18‒8 μm long on midbody (Figs 11D and 13B and 13C), and 19‒8 μm long on posterior body (Figs 11I and 13D). Dorsal simple chaetae present on all chaetigers (Table 3), slightly sigmoid, distally bifid with unequal sizes, “ypsiloid”, spinulated, with short spines reaching between tips (Figs 11F–13G, 13C, 13E and 13F). Ventral simple chaetae present from midbody onwards (Table 3), about as thick as falciger shafts, strongly sigmoid, smooth and bidentate, subdistal tooth slightly larger than distal one, with wide angle inbetween (Figs 11H and 13G). Parapodia from first few chaetigers with up to three aciculae each, progressively diminishing to single acicula per parapodium from midbody onwards; on anterior body, each parapodium with one acicula straight, with rounded tip, usually protruding from parapodial lobe, another subdistally bent and, occasionally, a third acicula, much thinner, straight (Fig 11C); on mid- and posterior body, aciculae subdistally inflated and curved, twisted, with tapered tip pointing upwards (Fig 11E and 11J). Pygidium with pair of thin anal cirri about twice as long as posterior body dorsal cirri. Pharynx through 4‒5 segments (Figs 6B, 11A and Table 3), anterior margin smooth (Fig 12B and 12D); pharyngeal tooth ovate, at about ⅔ of pharynx; proventricle through 4.5‒5.5 segments, with 26‒29 muscle cell rows (Figs 6B and 11A and Table 3).

#### Remarks

*Salvatoria ypsiloides* **n. sp.** is characterized by having palps shorter than prostomium; median antenna inserted between posterior pair of eyes; dorsal cirri elongate, digitiform, almost of uniform width along their length and distally hollow in appearance, present on all chaetigers; tooth at about ⅔ of the pharynx; falcigers with strongly bidentate blades, with wide angle between teeth, except for dorsalmost chaetae in each fascicle, which teeth are closer to each other; dorsal simple chaetae present from anterior body, distally bifid, with “ypsiloid” shape; and ventral simple chaetae sigmoid and bidentate, with subdistal tooth slightly larger than distal one. *Salvatoria euritmica*, *S*. *nitidula* and *S*. *rugulosa* (Verrill, 1900) [18] are the most similar species to *S*. *ypsiloides* **n. sp.**, in overall body shape or by sharing some of the above features. However, *S*. *ypsiloides* **n. sp.** differs from all congeners by the apparently distally hollow dorsal cirri and by the unique shape of the dorsal simple chaetae, looking ‘ypsiloid’.

*Salvatoria euritmica* and *S*. *nitidula* resemble *S*. *ypsiloides* **n. sp.** by possessing falcigers with strongly bidentate, spinulated blades, but both differ from *S*. *ypsiloides* **n. sp.** in having palps slightly longer than the prostomium; antennae and dorsal cirri throughout larger and rather spindle-shaped (although only slightly tapered distally in *S*. *nitidula*); pharyngeal tooth inserted near anterior margin of pharynx; blades of falcigers with narrower angle between teeth than in *S*. *ypsiloides* **n. sp.,** and with shorter spinulation in the dorsalmost blades; dorsal simple chaetae present from midbody (from anterior body in *S*. *nitidula*), bidentate, with short teeth and short spines; and ventral simple chaetae bidentate, with teeth similar to those of the falcigers [7, 17, 19].

*Salvatoria rugulosa* was described from Bermuda [18] and has been recorded from Florida, Gulf of Mexico and Cuba [19]; it resembles *S*. *ypsiloides* **n. sp.** in having the pharyngeal tooth located away from the anterior margin of pharynx and by having falcigers with bidentate, spinulated blades. In contraposition, *S*. *rugulosa* differs from *S*. *ypsiloides* **n. sp.** in having conical to triangular palps; short, spindle-shaped antennae and dorsal cirri throughout; all blades of falcigers with teeth closer to each other than in *S*. *ypsiloides* **n. sp.**; dorsal simple chaetae thin, bidentate, with teeth similar in size; and ventral simple chaetae less sigmoid, with teeth similar in size [18, 16].

#### Type locality

Fernando de Noronha Island, Pernambuco, Brazil (SW Atlantic).

#### Distribution

South Atlantic: NE Brazil (state of Pernambuco: Fernando de Noronha Archipelago), SE Brazil (states of Rio de Janeiro–Campos Basin–and Espírito Santo–Espírito Santo basin) (Fig 1A, 1C and 1D).

#### Etymology

The specific name refers to the characteristic “ypsiloid” appearance of the dorsal simple chaetae.

***Salvatoria* sp.**

**Fig 14:**
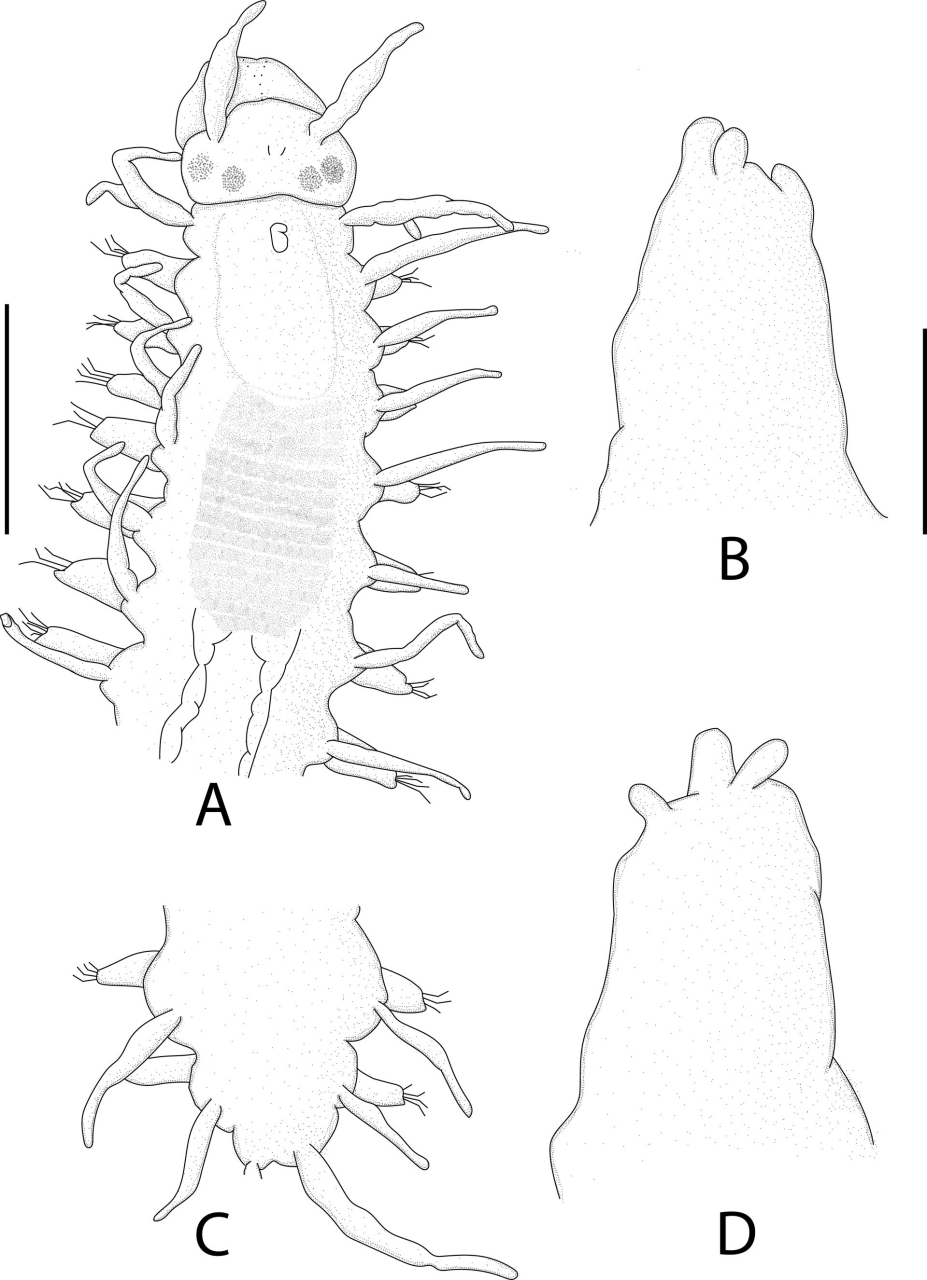
*Salvatoria* sp. (A). Anterior body, dorsal view; (B). Parapodial lobe from chaetiger 1; (C). Posterior end, dorsal view; (D). Parapodial lobe from chaetiger 8. Scale bars: A, C, 0.14 mm; B, D, 27 μm.

**Fig 15:**
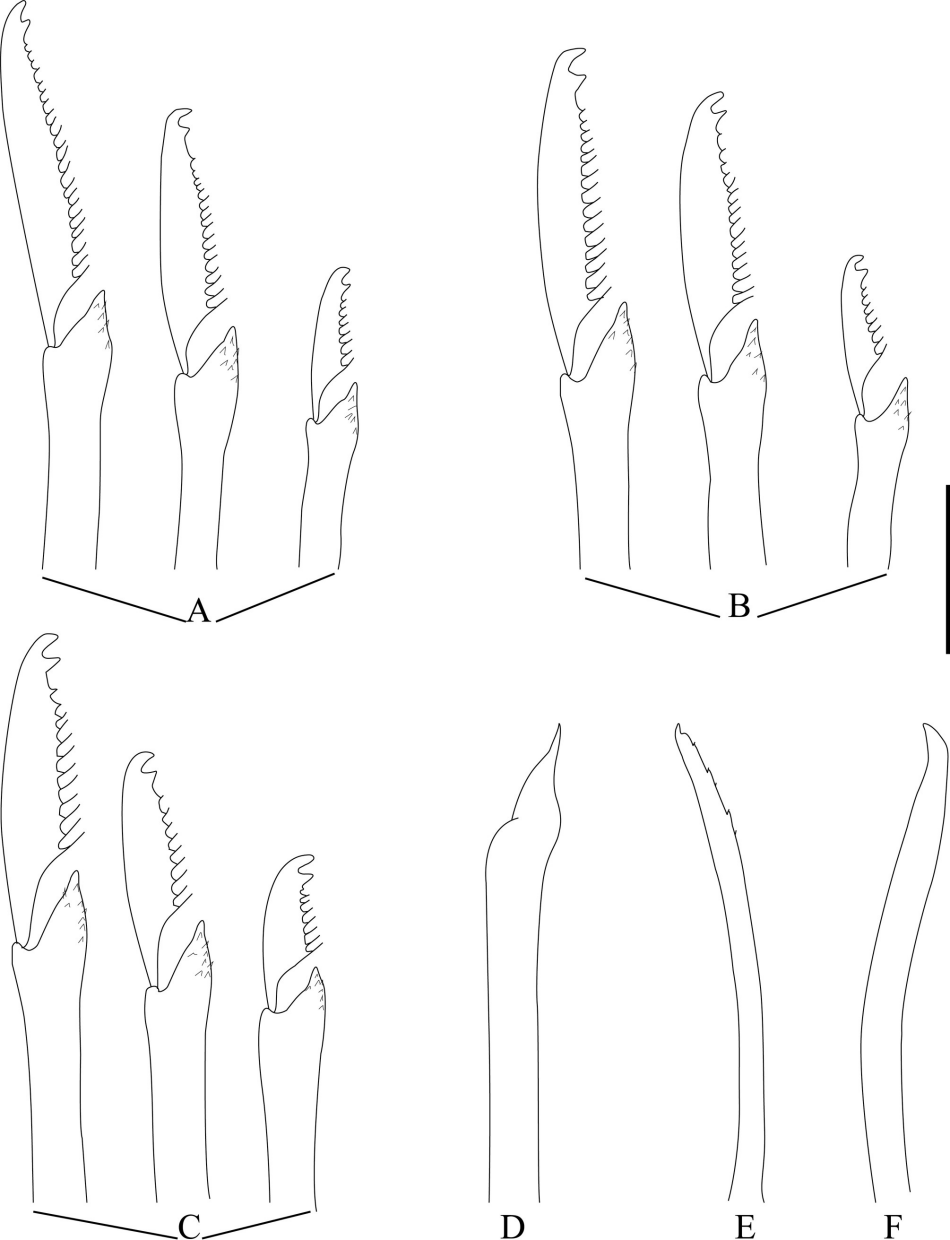
*Salvatoria* sp. (A–C). Falcigers, anterior, mid- and posterior body, respectively; (D). Acicula, posterior body; (E). Dorsal simple chaeta, midbody; (F). Ventral simple chaeta, posterior body. Scale bar: A–F, 7 μm.

Figs 14 and 15 and Table 1.

#### Material examined

Project ‘*Island Syllids*’—Fernando de Noronha Island, Buraco da Raquel (03°50’11”S 32°24’34”W), 1 m deep, in association with the sponge *Plakortis insularis*: 1 specimen (MZUSP 4115), coll. 23 April 2016. **Description.** Small-sized body, complete, 1.3 mm long, 0.14 wide, with 20 chaetigers. Palps slightly shorter than prostomium, reniform to triangular, dorsally completely united by membrane (Fig 14A). Prostomium subpentagonal (Fig 14A), with two pairs of eyes in open trapezoidal arrangement (Fig 14A). Antennae spindle-shaped; median antenna lost, scar indicating insertion between anterior pair of eyes; lateral antennae almost as long as palps and prostomium together, inserted in front of anterior pair of eyes, slightly away from anterior margin of prostomium (Fig 14A). Peristomium with two pairs of peristomial cirri, dorsal peristomial cirri about same length as lateral antennae, ventral peristomial cirri about half length of dorsal ones (Fig 14A). Dorsal cirri distally tapered, with bases slightly thickened, present on all chaetigers; dorsal cirri of chaetiger 1 longer than remaining, slightly exceeding width of segment; remaining dorsal cirri shorter than segment width; from chaetiger 2 onwards, cirri alternating in length, without defined pattern (Fig 14A). Ventral cirri digitiform, shorter than parapodial lobes. Parapodial lobes conical, about ¼ of corresponding segment width, distally with 2–3 papillae (Fig 14B and 14D). Parapodia with 6‒7 falcigers each, on anterior body and midbody chaetigers, 3 falcigers on each posterior body parapodium; falcigers with shafts slightly spinulated subdistally; falciger blades spinulated, with coarse spines directed slightly upwards, and strongly bidentate, distal tooth slightly larger than subdistal one (Fig 15A–15C); blades with dorsoventral gradation in length, measuring 22‒7 μm long on anterior body (Fig 15A), 23‒7 μm long on midbody (Fig 15B), and 20‒6 μm long on posterior body (Fig 15C). Dorsal simple chaetae present from second chaetiger onwards, thinner than falciger shafts, slightly sigmoid, unidentate, spinulated, serrate in appearance (Fig 15E). Ventral simple chaetae present only on two posteriormost chaetigers, thicker than dorsal simple chaetae, although not as thick as falciger shafts, sigmoid, unidentate, apparently smooth (Fig 15F). Single acicula per parapodium throughout, subdistally inflated and curved, with acute tip pointing upwards (Fig 15D). Pygidium with pair of anal cirri slightly longer than dorsal cirri of chaetiger 1 (Fig 14C). Pharynx through 3.5 segments, anterior margin apparently smooth; pharyngeal tooth rhomboidal, located slightly away from anterior margin of pharynx; proventricle through ~3 segments, with ~15 muscle cell rows (Fig 14A).

#### Remarks

As we found a single specimen of *Salvatoria* sp., we preferred not to investigate the specimen under SEM, what made it difficult to discern more detailed characters. Furthermore, the median antenna, which is an important taxonomic character, is missing in this specimen, precluding more specific identifications. The specimen was found with the sponge *Plakortis insularis* Moraes & Muricy, 2003 [38], without information whether the specimen was over or within the sponge.

The specimen presents some similarities with *S*. *clavata* from the Iberian peninsula, described by [7], in the overall morphology of the antennae and dorsal cirri, in the presence of characteristic distal papillae on parapodial lobes and, to some extent, in the morphology of the falciger blades. However, *S*. *clavata* differs from *Salvatoria* sp. for being larger and wider, 3.5 mm long, 0.35 mm wide, with 35 chaetigers; by having shorter parapodial lobes; more falcigers per parapodium, 10 chaetae on anterior body, 7–4 on posterior body; blades of falcigers with marked dorso-ventral gradation in length only in anterior parapodia, and with slightly shorter spines; both dorsal and ventral simple chaetae bidentate and spinulated; and proventricle with more muscle cell rows, 20–23.

San Martín [7] pointed out that there are at least three different forms registered as *S*. *clavata* in the Iberian peninsula, two by [39] and one by [40], each one deviating somewhat from the description presented by [7], who, nonetheless, considered them as intraspecific variations.

As mentioned above, *S*. cf. *nitidula* characterized herein is similar to *S*. *clavata* from the Iberian Peninsula reported by [7]. In comparison to *Salvatoria* sp., *S*. cf. *nitidula* differs by having longer and wider body, 2.37 mm long, 0.18 mm wide, with 31 chaetigers; palps as long as prostomium, with a distal notch; both dorsal and ventral simple chaetae bidentate and spinulated; and proventricle with more muscle cell rows, 16–20.

Other congeneric species similar to *Salvatoria* sp. is *S*. *marielleae* **n. sp.**, by the overall morphology of antennae, dorsal cirri throughout and falciger blades. Nevertheless, *Salvatoria* sp. has dorsal cirri larger and slightly thicker; parapodial lobes distally papillated; falciger blades with distal tooth slightly larger than the subdistal one throughout and with thicker spines; and both dorsal and ventral simple chaetae unidentate and less sigmoid.

#### Distribution

South Atlantic: NE Brazil (state of Pernambuco: Fernando de Noronha Island) (Fig 1A and 1D).

## Discussion

Five out of the 12 species reported herein are only known from Brazilian waters: *Salvatoria breviarticulata* **comb. nov.**, *S*. *longiarticulata* **comb. nov.**, *S*. *marielleae* **n. sp.**, *S*. *ypsiloides* **n. sp.** and *S*. *nitiduloides* **n. sp.**, in addition to the unidentified *Salvatoria* sp. Brazilian specimens of *Salvatoria* cf. *nitidula* and *S*. *neapolitana* were also described, with some subtle differences in comparison to their original descriptions. *Salvatoria clavata*, *S*. *euritmica*, *S*. *heterocirra* and *S*. *limbata* had previously been identified in Brazilian waters, but without voucher material, nor characterizations or illustrations provided [9, 20]; the four species were not examined for this work. Additionally, no material identified as Brazilian *S*. *limbata* is available as vouchers in collections (Table 1), so we consider this record as doubtful.

*Salvatoria clavata*, *S*. *euritmica*, *S*. *limbata* and *S*. *neapolitana* have very wide distributions: while their type-localities are in the Atlantic Ocean, they were also recorded in the Indian and Pacific Oceans (Fig 1A and Table 1). However, such distribution is not consistent with the low dispersal capabilities and the reproduction process present in the Exogoninae, which involves parental care. Virtually all species of the Syllidae previously considered cosmopolitan, when carefully studied with morphometrics and genetic data, were found to present much more restricted distributions [41–44], which warrants for some restrictions on the records of these species, specially outside the Atlantic. On the other hand, out of all these species, only *S*. *neapolitana* was examined herein; Brazilian specimens match almost completely the type specimens from the Mediterranean Sea, thus we assume the presence of this species in the coast of Brazil is correct.

All morphological characters in Table 1 were informative for the taxonomy of *Salvatoria*. The shape, size and insertion of the antennae in the prostomium as well as the shape and length of the peristomial and dorsal cirri are fundamental to diagnose and describe the new species presented in this work. It is worth mentioning that most species of the genus have antennae, peristomial and dorsal cirri spindle shaped or with subtle variations, such as spindle shaped to subulated or digitiform. However, *S*. *neapolitana* has conical, thickened antennae and cirri, and *S*. *opisthodentata* (Hartmann-Schröder, 1979) [45] and *S*. *ypsiloides* **n. sp.** have digitiform appendages. Additionally, S. *ypsiloides* **n. sp.** also shows some dorsal and ventral cirri distally hollow in appearance, unique in the genus.

For some species, antennae, peristomial and dorsal cirri have more or less the same shape, as in *S*. *euritmica*; in contraposition, for another group of species, such as in *S*. *mariellae* **n. sp.**, antennae and cirri have different shapes. Dorsal and ventral simple chaetae are also very informative. Here we report the Y-shaped dorsal simple chaetae and ventral simple chaetae with teeth widely open, both unique in the genus, present in *S*. *ypsiloides* **n. sp.**

A phylogenetic analysis, ideally combining different classes of data, such as morphological, molecular and ecological, may help to infer phylogeographic hypothesis and elucidate evolutionary routes within the genus.
