# Correction: A synopsis of *Salvatoria* McIntosh, 1885 (Annelida: Syllidae: Exogoninae) from Brazilian coastal and oceanic waters

**DOI:** 10.1371/journal.pone.0258022

**Published:** 2021-09-23

**Authors:** Rodolfo Leandro Nascimento, Marcelo Veronesi Fukuda, Karla Paresque, João Miguel de Matos Nogueira, Paulo Cesar de Paiva

There are errors in Table 1. Please see the correct Table 1 here.

**Table 1:** Taxonomic and biogeographic data on the species of *Salvatoria* recorded for Brazilian waters, with reference to voucher material. Type localities in bold. Data of *S*. *breviarticulata* and *S*. *longiarticulata* from [21], *S*. *limbata*, *S*. *clavata* and *S*. *euritmica* from [7] and [20].

	Original description	Brazilian material	Source of Records	Body length x width (mm) / nº of chaetigers	Palps	Antennae	Dorsal cirri	Parapodia	Number of falcigers (anterior / posterior)	Falciger blades morphology	Falciger blades length (anterior / posterior blades (µm)	Dorsal simple chaeta	Ventral simple chaeta	Number of pharynx segments / tooth position	Number of proventricle segments / muscle cell rows	Habitat	Distribution
*S*. *breviarticulata* comb. nov.	[21]	MHN-BPO 67/0. MHN-BPO 67/1–3	[9,20,21]	1.9 x 0.2 / 26	More than twice as long as prostomium, united up to mid-length	Spindle shaped	Same shape as antennae	Smooth	4 throughout	Bidentate, distal tooth larger throughout, oblique space inbetween	11–8 / 10 from midbody	Present from midbody; bidentate	Only on posterior parapodia, sigmoid; slightly bidentate	4 / at anterior margin	4 / 23	Coastal, subtidal zones, 4–73 m deep. Associated with *M*. *hispida* (Verrill, 1868) coral heads	Only known from type locality. Atlantic Ocean: Brazil (**São Paulo**).
*S*. *clavata*	[26]	MHN-BPO JN 14/1–4	[9,20]	3.5 x 0.35 / 31	As long as prostomium, almost completely united, leaving a distal notch	Spindle shaped	Same shape as antennae	2–3 papillae	10 / 4–7	Bidentate, distal tooth larger throughout, oblique space inbetween	19–12 / 12–11	Present from first chaetiger; bidentate	Only on posterior parapodia; straight, bidentate	3–4 / at first ⅓	3–4 / 20–23	Coastal and oceanic, intertidal to 46 m deep. Associated with algae, corals, bryozoans, calcareous algae, but also found in soft sediments	Atlantic Ocean: Norwegian Sea, **Normandy**, Mediterranean Sea, USA, Cuba, Brazil (São Paulo). Pacific Ocean: Sea of Japan.
*S*. *euritmica*	[27]	MHN-BPO JN 16/1–3	[9,20]	4 x 0.35 / 29	As long as prostomium, united up to mid-length	Spindle shaped	Same shape as antennae	2–3 papillae	12 / 5–6	Bidentate, teeth similar in size, rounded space inbetween	31–20 / 12–9	Present from midbody; bidentate	Only on posterior parapodia; sigmoid, bidentate	3–4 / slightly away from anterior margin	~4 / 15–20	Coastal and oceanic, intertidal to 44 m deep. Associated with corals, algae, and seagrass, but also found in soft sediments	Atlantic Ocean: Southern Mediterranean Sea (**Gibraltar**), Caribbean Sea, Brazil (São Paulo). Pacific Ocean: Australia.
*S*. *heterocirra*	[28]	MHN-BPO JN 15/1–25	[9, 20, 24]	? x 0.9 /??	Longer than prostomium, almost completely united, leaving a distal notch	Spindle shaped	Different from antennae, spindle shaped to subulate	Smooth	??	Bidentate, distal teeth similar in size throughout, oblique space inbetween	18–8 throughout	Bidentate	Slightly sigmoid, bidentate.	~4 / slightly away from anterior margin	2–2.5 / 28	Coastal and oceanic, intertidal to 20 m deep. Associated with corals, but also found in soft sediments	Atlantic Ocean: Gulf of Mexico, Brazil (São Paulo). Pacific Ocean: **Mexico**, Costa Rica, Panamá.
*S*. *limbata*	[29]	Without material deposited	[9]. Doubtful record	2 x 0.15 / 30	Shorter than prostomium, almost completely united, leaving a distal notch	Spindle shaped	Different from antennae, spindle shaped to subulate	2–3 papillae	10–9 / 6–7	Unidentate	24–14 / —	Present from first chaetiger; unidentate	Only on posterior parapodia; curved, unidentate	~3 / slightly away from anterior margin	~3 / 16–18	Coastal and oceanic, intertidal to 144 m deep. Associated with algae, corals, sponges, but also found in soft sediments	Atlantic Ocean: Mediterranean Sea (**France**), Caribbean Sea, Brazil (São Paulo), Argentina. Pacific Ocean: Mexico, Chile, Australia.
*S*. *longiarticulata* comb. nov.	[21]	MHN-BPO 68/0. MHN-BPO 68/1–5.	[9, 20, 21, 30], this paper	2.47 x 0.17 / 28	Shorter than prostomium, almost completely united, leaving a distal notch	Spindle shaped	Different from antennae, spindle shaped to subulated	Smooth	6 throughout	Bidentate, distal tooth longer, subdistal tooth very slender; oblique space inbetween	34–18 throughout	Present from anterior body; unidentate	Only on posterior body; sigmoid, bidentate	3–4 / slightly away from anterior margin	3–4 / 24	Coastal, intertidal to 7 m deep. On rocky shores and associated with *M*. *hispida* coral heads	Atlantic Ocean: Brazil (Paraíba, Pernambuco, **São Paulo**)
*S*. *marielleae* n. sp.	This paper	MNRJP and MZUSP (see below)	This paper	2.3 x 0.18 / 22	As long as prostomium, almost completely united, leaving a distal notch	Spindle shaped	Different from antennae, distally tapered, with bases slightly thickened	Smooth	7–5 throughout	Bidentate, teeth similar in size; rounded space inbetween	21‒9 / 20‒10	Present from proventricle level; bidentate	Only on posterior parapodia; sigmoid, bidentate	3 / at anterior margin	~2.5 / 18‒19	Oceanic, intertidal to 12 m deep. Associated with red algae	Atlantic Ocean: Brazil (Pernambuco–**Fernando de Noronha island)**
*S*. *neapolitana*	[23]	MZUSP (see below)	[30], this paper	1.8 x 0.15 / 23	Longer than prostomium, almost completely united, leaving a distal notch	Conical, elongated, thick, distally pointed	Same shape as antennae	1 papilla	10 / 2–5	Sub-bidentate, subdistal tooth thin, spine-like; oblique space inbetween	19–9 throughout	Present from first chaetiger; unidentate	Only on posterior parapodia; slightly curved, unidentate	3 / at first ⅓	3.5 / 20	Coastal and oceanic, intertidal to 19 m deep. Associated with algae, but also found in soft sediments	Atlantic Ocean: Mediterranean Sea, France, Spain, **Italy (Gulf of Naples**), Canary Islands, Venezuela, Caribbean Sea, Brazil (São Paulo). Pacific Ocean: Australia
*S*. cf. *nitidula*	[18]	MZUSP (see below)	[30], this paper	2.37 x 0.18 / 31	As long as prostomium, almost completely united, leaving a distal notch	Spindle shaped to subulated	Same shape as antennae	Smooth	7–6 throughout	Bidentate, distal tooth slightly larger on anterior and midbody, teeth about same size on posterior body; oblique space inbetween	30–10 / 25–10	Present from first chaetiger; bidentate distal tooth slightly longer	Only on posterior parapodia; sigmoid, bidentate, smooth	3.5–4 / slightly away from anterior margin	3–4.5 / 16–20	Coastal, intertidal. Associated with algae and similar substrates on rocky shores	Records of original description–Atlantic Ocean: **Bermuda;** Caribbean Sea (Gulf of Mexico, Cuba). Current records: SE Brazil (São Paulo)
*S*. *nitiduloides* n. sp.	This paper	MZUSP (see below)	This paper	2.2 x 0.2 / 29	As long as prostomium, almost completely united, leaving a distal notch	Spindle shaped to subulated	Same shape as antennae	Smooth	10–7 / 3–4	Bidentate, distal tooth slightly larger throughout; oblique space inbetween	22–8 / 24–11	Present from anterior body; bidentate, teeth similar in size	Only on posterior parapodia; sigmoid, bidentate, spinulated	3–4 / slightly away from anterior margin	2–3 / 19–22	Coastal, intertidal. Associated with algae and similar substrates on rocky shores	Atlantic Ocean: Brazil (Paraíba, Pernambuco**–Ilha de Itamaracá**)
*S*. *ypsiloides* n. sp.	This paper	MNRJP and MZUSP (see below)	This paper	2.2 x 0.12 / 32	Shorter than prostomium, almost completely united, leaving a distal notch	Spindle shaped to digitiform	Different from antennae, elongate, digitiform, sometimes slightly swollen distally hollow	Smooth	7–5 throughout	Bidentate, distal tooth slightly shorter on anterior body; teeth about same size on mid- and posterior body; wide angle inbetween	18‒7.4 / 19‒8	Present from first chaetiger; bifid, “ypsiloid” in appearance	From midbody parapodia; sigmoid, bidentate	4–5 / slightly away from anterior margin	4.5‒5.5 / 26‒29	Coastal and oceanic, subtidal to 970 m deep. Associated with red algae, soft sediments	Atlantic Ocean: Brazil (Pernambuco–**Fernando de Noronha island**, Espírito Santo, Rio de Janeiro)
*Salvatoria* sp.	This paper	MZUSP (see below)	This paper	1.3 x 0.14 / 20	Shorter than prostomium, completely united	Spindle shaped	Different from antennae, distally tapered, with bases slightly thickened	2–3 papillae	7‒6 / 3	Bidentate, distal tooth slightly larger; oblique space inbetween	22‒7 / 20‒6	Present from chaetiger 2; unidentate	Only on posterior body; sigmoid, unidentate	3.5 / slightly away from anterior margin	~3 / 15	Oceanic, intertidal to 1 m deep. Associated with sponge *Plakortis insularis*	Atlantic Ocean: Brazil (Pernambuco–Fernando de Noronha)
